# Regulation of the firing activity by PKA‐PKC‐Src family kinases in cultured neurons of hypothalamic arcuate nucleus

**DOI:** 10.1002/jnr.24516

**Published:** 2019-08-12

**Authors:** Xiao‐Dong Sun, Anqi Wang, Peng Ma, Shan Gong, Jin Tao, Xian‐Min Yu, Xinghong Jiang

**Affiliations:** ^1^ Key Laboratory of Pain Basic Research and Clinical Therapy, Department of Physiology and Neurobiology Medical College of Soochow University Suzhou China

**Keywords:** action potential firing activity, hypothalamic arcuate nucleus, pain, phosphorylation, protein kinase A, protein kinase C, RRID:AB_10015289, RRID:AB_10865528, RRID:AB_141607, RRID:AB_1524202, RRID:AB_2168219, RRID:AB_2284224, RRID:AB_2298772, RRID:AB_2313567, RRID:AB_2535792, RRID:AB_2687938, RRID:AB_2750616, RRID:AB_777294, RRID:AB_779042, RRID:SCR_007369, RRID:SCR_014329, Src family kinases

## Abstract

The cAMP‐dependent protein kinase A family (PKAs), protein kinase C family (PKCs), and Src family kinases (SFKs) are found to play important roles in pain hypersensitivity. However, more detailed investigations are still needed in order to understand the mechanisms underlying the actions of PKAs, PKCs, and SFKs. Neurons in the hypothalamic arcuate nucleus (ARC) are found to be involved in the regulation of pain hypersensitivity. Here we report that the action potential (AP) firing activity of ARC neurons in culture was up‐regulated by application of the adenylate cyclase activator forskolin or the PKC activator PMA, and that the forskolin or PMA application‐induced up‐regulation of AP firing activity could be blocked by pre‐application of the SFK inhibitor PP2. SFK activation also up‐regulated the AP firing activity and this effect could be prevented by pre‐application of the inhibitors of PKCs, but not of PKAs. Furthermore, we identified that forskolin or PMA application caused increases in the phosphorylation not only in PKAs at T197 or PKCs at S660 and PKCα/βII at T638/641, but also in SFKs at Y416. The forskolin or PMA application‐induced increase in the phosphorylation of PKAs or PKCs was not affected by pre‐treatment with PP2. The regulations of the SFK and AP firing activities by PKCs were independent upon the translocation of either PKCα or PKCβII. Thus, it is demonstrated that PKAs may act as an upstream factor(s) to enhance SFKs while PKCs and SFKs interact reciprocally, and thereby up‐regulate the AP firing activity in hypothalamic ARC neurons.

## Graphical abstract

The present study has demonstrated that PKAs may act as an upstream factor(s) to enhance Src family kinases (SFKs) while PKCs and SFKs may interact reciprocally, and thereby up‐regulate the action potential firing activity of neurons in the hypothalamic arcuate nucleus.

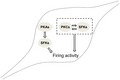

**Significance**

The hypothalamic arcuate nucleus (ARC) has been found to be involved in the control of pain hypersensitivity. In order to better understand the actions of PKAs, PKCs, and SFKs in the regulation of neuronal activity, we investigated the regulation of the action potential (AP) firing activity by these kinases in cultured ARC neurons. This work demonstrates that PKAs may act as an upstream factor(s) to enhance SFKs while PKCs and SFKs may interact reciprocally, and thereby up‐regulate the AP firing activity in hypothalamic ARC neurons.

## 1 INTRODUCTION

The cAMP‐dependent protein kinase A family (PKAs) (Dina, Chen, Reichling, & Levine, 2001; Gu, Wang, Li, & Huang, 2016; Hu & Gereau, 2003; Kawasaki et al., 2004; Wang, Ruan, Hong, Chabot, & Quirion, 2013), protein kinase C family (PKCs) (Gu et al., 2016; Guo et al., 2004; He & Wang, 2015; Kawasaki et al., 2004; Zhu, Xu, Cuascut, Hall, & Oxford, 2007), and Src family kinases (SFKs) (Guo et al., 2006; Kawasaki et al., 2004; Mickle, Shepherd, Loo, & Mohapatra, 2015) have been found to play important roles in the regulation of neuroplasticity that is associated with the formation and persistence of pain hypersensitivity. The effects of PKA or PKC activation either alone or together in spinal dorsal horn neurons can be reduced by the inhibition of Src (Kawasaki et al., 2004). However, conflicting data were also reported. For example, PKAs were found to activate (Sun, Ke, & Budde, 1997; Yang et al., 2011; Yeo et al., 2011) or inhibit (Abrahamsen, Vang, & Tasken, 2003; Trepanier, Lei, Xie, & MacDonald, 2013; Vang et al., 2001; Yaqub et al., 2003) SFKs. While it was found that PKCs might enhance N‐methyl‐D‐aspartate receptor (NMDAR) activity via activating SFKs (Grosshans & Browning, 2001; Lu, Kojima, et al., 1999), it had also been reported that PKCs might reduce the activity of NMDARs through directly phosphorylating NMDARs, which caused increases in the Ca^2+^‐dependent inactivation of the receptors (Lu, Jackson, Bai, Orser, & MacDonald, 2000; MacDonald, Kotecha, Lu, & Jackson, 2001).

Neurons in the hypothalamic arcuate nucleus (ARC) are found to be involved in the descending modulation of nociception (Bach, 1997; Sim & Joseph, 1989, 1991; Wang et al., 2015; Yin, DuanMu, Guo, Yu, & Zhang, 1984). Recent studies (Bu et al., 2015; Peng et al., 2011; Xu et al., 2012; Zheng et al., 2016) have shown that with the development of visceral or peripheral inflammation, increases in the neuronal discharge activity and the expression of active PKCs, SFKs, and the phosphorylated GluN2B subunit at Y1472 occur in the ARC area. Application of inhibitors of PKCs or SFKs into the ARC area blocks the enhancement of the expression of active PKCs, SFKs, and the phosphorylation of GluN2B at Y1472 in this area, and also attenuates the inflammation‐induced enhancement of the discharge activity of ARC neurons and pain hypersensitivity (Bu et al., 2015; Peng et al., 2011; Xu et al., 2012; Zheng et al., 2016). Furthermore, we have identified that Src, but not Fyn or Lyn in SFKs, is activated following the development of peripheral inflammation induced by the injection of complete Freund's adjuvant (CFA) into the hind pawl of rats (Ma et al., 2019). It is found that the knockdown of Src in the ARC area blocks the increases in the expression of activated SFKs and the phosphorylation of GluN2B subunit at Y1472, and reduces pain hypersensitivity induced by the CFA injection (Ma et al., 2019).

Potential functional interactions among PKAs, PKCs, and SFKs in the regulation of pain hypersensitivity have been implicated in the spinal cord dorsal horn (Guo et al., 2004; Kawasaki et al., 2004), rostral ventromedial medulla (Guo et al., 2006), and ARC (Bu et al., 2015; Xu et al., 2012; Zheng et al., 2016). Characterizing the functional interactions among these molecules in the CNS is still required in order to understand the detailed mechanisms underlying the regulation of pain hypersensitivity. Therefore, we did this work as the first step, in cultured neurons isolated from the ARC area of rats, to identify the actions of PKCs, PKAs, and SFKs in regulating the action potential (AP) firing activity.

## 2 MATERIAL AND METHODS

### 2.1 ARC neuron culture

ARC tissues isolated acutely from 1‐day‐old Sprague‐Dawley rat pups (both male and female) were used for culture. Total 420 rat pups were used. They were obtained from an in‐house breeding colony with parent animals from the laboratory animal center at Soochow University. Animal care and experimental procedures were conducted following the guidelines of Animal Care and Use Committee of the Medical College of Soochow University and approved by Ethics Committee of Soochow University in accordance with the guidelines of the International Association for the Study of Pain. Animals were housed on a 12‐hr light/dark cycle. The pups were reared in large cages by their mothers with free access to food and water.

Studies dealing with sex differences, which may produce biological variables, were not performed in this work. Following decapitation the whole brain was quickly removed and transferred to a 100 mm dish filled with ice‐cold Hank's balanced salt solution (HBSS, pH 7.4; Gibco, Shanghai, China). Coronal brain sections (≈ 500 μm thick) were performed throughout the hypothalamic region under a stereomicroscope (SMZ455, Olympus, Tokyo, Japan). Tissues of the ARC region (Paxinos & Watson, 2007) (see Figure S1) were then dissected and cut. After wash for twice with HBSS the ARC tissues were treated with papain (2 mg/ml) dissolved in Neurobasal‐A medium containing 5 mM of L‐cystein (pH 7.4) for 25 min at 37°C. Following gentle mechanical trituration in Neurobasal‐A medium supplemented with 10% of FBS (Gibco), 0.2% of insulin (Gibco), and 1% of Gluta‐max (containing 2 mM of L‐alanyl‐L‐glutamine dipeptide) (Gibco), the tissues were filtered with Cell Strainer (70 µM, BD, NY, USA). Dissociated ARC cells (10^5^ cells per ml) were plated onto poly‐L‐lysine coated glass coverslips (diameter: 12 mm) and cultured in humidified air with 5% CO_2_ at 37°C for 4–6 days with Neurobasal‐A medium supplemented with 2% of B27 and 1% of Gluta‐max before use for experiments.

### 2.2 Electrophysiological recording

Whole cell recordings in the current clamp model were performed for recording current injection‐induced APs, as described previously (Wang et al., 2011). In brief, ARC cultures were placed in a recording chamber on an inverted microscope (Ti‐DH, Nikon, Tokyo, Japan) equipped with a 40× Varel Relief Contrast System. Recorded cells were monitored during experiments to confirm that the same cells were recorded before and after any treatment. The cultures were continuously perfused with a standard external solution (0.5 ml/min) containing (in mM) 128 NaCl, 2 KCl, 2 CaCl_2_, 2 MgCl_2_, 30 glucose, 25 HEPES, pH was adjusted to 7.4 with NaOH, osmolarity: 305 mOsm. Recording electrodes pulled from filamented borosilicate glass (Sutter Instruments, Novato, CA) were fire‐polished, and filled with an internal solution composed of (in mM): 110 KCl, 10 NaCl, 2 EGTA, 25 HEPES, 4 Mg‐ATP, and 0.3 Na_2_GTP, pH was adjusted to 7.3 with KOH, osmolarity: 295 mOsm. The DC resistance of recording electrodes was 7.3 ± 1.6 MΩ (mean ± *SD*). Recordings were performed at room temperature (23 ± 1°C) with a MultiClamp 700B amplifier (Molecular Devices, San Jose, CA). Electrical signals filtered at 1 kHz were recorded through the amplifier following the subtraction of the capacitive transients and digitized at 10 kHz. Off‐line analysis of recorded electrical signals was conducted using the Clampex 10.2 (Molecular Devices). All recorded neurons were clamped at −60 mV. AP firing was induced by injection of depolarizing current pulses (increasing step amplitude: 10 pA; duration: 1 s; injection interval: 600 ms).

### 2.3 Western blot analysis

Western blotting experiments were performed as described previously (Lei et al., 2002; Xu et al., 2008; Zheng et al., 2016). In brief, ARC cultures were washed three times with ice‐cold PBS and scraped into ice‐cold RIPA buffer (Beyotime Biotechnology, Shanghai, China) supplemented with EDTA‐free cocktails of protease and phosphatase inhibitors (Roche, Basel, Switzerland), DTT (0.5 mM, Beyotime Biotechnology, Shanghai, China) and PMSF (1 mM, Beyotime Biotechnology). ARC cells were lysed via sonication. The homogenates were then centrifuged at 12,000*g* for 20 min at 4°C. Samples subjected to SDS‐PAGE were generated by adding one‐third of 4× loading buffer (Thermo, Shanghai, China).

BCA assay (Pierce, Shanghai, China) was used to determine protein concentrations. Each lane of gels was loaded with 50 μg of sampled proteins. After the proteins were transferred to 0.45 µm PVDF membranes (Millipore, Shanghai, China), the membrane was cropped, and then stripped and successively probed with primary antibodies (overnight at 4°C) including phospho‐PKAα/β/γ antibody (pT197, RRID:AB_1524202), phospho‐PKC antibody (pan, pS660. RRID:AB_2168219), phospho‐PKCα/βII antibody (pT638/641, RRID:AB_2284224), phospho‐SFK antibody (pY416, RRID:AB_10860257), PKAα/β/γ antibody (RRID:AB_2750616), PKCα antibody (RRID:AB_777294), PKCβII antibody (RRID:AB_779042), Src antibody (RRID:AB_10865528), and β‐actin antibody (RRID:AB_2687938). Detailed information about these antibodies is reported in Table 1. A secondary antibody, peroxidase‐conjugated goat anti‐rabbit IgG (RRID:AB_2313567) or peroxidase‐conjugated goat anti‐mouse IgG (RRID:AB_10015289) (for detailed information see Table 1) was utilized to visualize the primary antibody staining. Stripping was considered to be successful if no specific staining signal could be noted by the incubation of the stripped membrane with a secondary antibody. Examples of blots from same full length PVDF membranes, which were stripped and successively probed with antibodies, are shown in Figure S2.

**Table 1:** Antibody reporting

Ab Name	Immunogen	Manufacture				Concentration
		Name	Species	Cat. number	RRID	
Anti‐PKA alpha/beta/gamma (catalytic subunit) (phospho T197) antibody	A synthesized peptide corresponding to aa 150–250 containing phosphorylated Thr197 in the catalytic subunit of human PKAα/β/γ	Abcam	Rabbit, monoclonal	ab75991	AB_1524202	1:10,000 w
Phospho‐PKC (pan) (βII Ser660) rabbit Ab	A synthesized peptide corresponding to residues surrounding phosphorylated Ser660 of human PKC βII	CST	Rabbit, polyclonal	9371s	AB_2168219	1:1,000 w
Phospho‐PKCα/βII (Thr638/641) rabbit Ab	A synthesized peptide corresponding to residues surrounding phosphorylated Thr638/641 of human PKCα/βII	CST	Rabbit, polyclonal	9375s	AB_2284224	1:1,000 w
Phospho‐Src Family (Tyr416) (D49G4) rabbit mAb	A synthesized peptide corresponding to residues surrounding phosphorylated Tyr419 of human Src protein	CST	Rabbit, monoclonal	6943s	AB_10860257	1:1,000 w
PKAα/β/γ cat polyclonal antibody	A Synthesized peptide corresponding to aa 166–215 of human PKAα/β/γ CAT	ImmunoWay	Rabbit, polyclonal	YT3749	AB_2750616	1:500 w
Anti‐PKC alpha antibody	A synthesized peptide corresponding to aa 650 to the end of C‐terminus of human PKCα	Abcam	Rabbit, monoclonal	ab32376	AB_777294	1:1,000 w
						1:500 Fl image
Anti‐PKC beta 2 antibody	A synthesized peptide corresponding to aa 600–700 of human PKCβII	Abcam	Rabbit, monoclonal	ab32026	AB_779042	1:1,000 w
						1:250 Fl image
Anti‐Src antibody	A synthesized peptide corresponding to aa 1–100 of human Src	Abcam	Rabbit, monoclonal	ab109381	AB_10865528	1:10,000 w
Beta Actin Antibody	Fusion Protein	Proteintech	Mouse, monoclonal	66009‐1‐Ig	AB_2687938	1:5,000 w
Peroxidase AffiniPure goat anti‐rabbit IgG (H + L)	Rabbit IgG(H + L)	Jackson	Goat, polyclonal	111‐035‐003	AB_2313567	1:5,000 w
Peroxidase AffiniPure goat anti‐mouse IgG (H + L)	Mouse IgG(H + L)	Jackson	Goat, polyclonal	115‐035‐003	AB_10015289	1:5,000 w
Anti‐NeuN antibody	Purified cell nuclei from mouse brain	Merck Millipore	Mouse, monoclonal	MAB377	AB_2298772	1:250 Fl image
Donkey anti‐rabbit IgG (H + L) highly cross‐adsorbed secondary antibody, Alexa Fluor 488	Gamma Immunoglobins Heavy and Light chains	Thermo	Donkey, polyclonal	A21206	AB_2535792	1:1,000 Fl image
Donkey anti‐mouse IgG (H + L) highly cross‐adsorbed secondary antibody, Alexa Fluor 488	Gamma Immunoglobins Heavy and Light chains	Thermo	Donkey, polyclonal	A21202	AB_141607	1:1,000 Fl image

Abbreviations: Fl image: For immunofluorescent staining; W: For Western blotting.

Samples from cultured cells without any treatment (labeled as “naïve” in figures and following text) were examined in each (or each repeat) biochemical experiment in order to control variations which may occur from one experiment to another. Densitometry analysis of all western blots was conducted and the ratio of the band intensity versus that of β‐actin was calculated and then normalized to the ratio detected in samples from naïve cells. The normalized ratios were used to show the effects of any treatment. At least five replicates were performed for the Western blot.

### 2.4 Drug application

Effects of KT5720 (KT, 3 μM; Tocris, Bristol, UK), GF109203X (GF, 5 μM), chelerythrine chloride (CC, 10 μM; Millipore), 4‐Amino‐5‐(4‐chlorophenyl)‐7‐(t‐butyl)pyrazolo[3,4‐d]pyrimidine (PP2; 10 μM), 4‐Amino‐7‐phenylpyrazol[3,4‐d]pyrimidine (PP3, 10 μM; Millipore), forskolin (FSK; 1, 10, 50, 100 μM) co‐applied with 3‐Isobutyl‐1‐methylxanthine (IBMX, 50 μM), Phorbol 12‐myristate 13‐acetate (PMA, 0.1, 1, 5 10 μM), EPQ(pY)EEIPIA (1 mM; Sangon Biotech, Shanghai, China), and EPQYEEIPIA (1 mM; Sangon Biotech) were examined in this work. Each of these agents were prepared as concentrated stock solutions, and then diluted to final concentrations (1:1,000 or more) with the standard external (Vehicle) or internal solution for immediate use. All chemicals/drugs used in this study were purchased from Sigma (Shanghai, China) except where as indicated.

### 2.5 Immunofluorescence image

For immunofluorescence studies of ARC neurons, cultured cells were fixed and permeabilized by treatment with 4% of polyoxymethylene for 30 min and 0.15% of Triton for 30 min, and then double labeled with NeuN antibody (RRID:AB_2298772; see Table 1) and DAPI. The NeuN antibody staining was visualized by incubation with an Alexa Fluor Donkey anti‐mouse IgG (H + L) highly cross‐adsorbed secondary antibody (RRID:AB_141607; see Table 1).

For studies of PKCα and PKCβII translocations, after treatment with drugs as indicated ARC neurons were fixed and permeabilized as motioned above and then incubated with an antibody of PKCα (RRID:AB_777294) or PKCβII (RRID:AB_779042) (see Table 1). The PKCα or PKCβII antibody staining was then visualized by incubation with an Alexa Fluor 488‐labeled goat anti‐rabbit secondary antibody (RRID:AB_2535792; see Table 1). Fluorescence images were captured under an inverted fluorescence microscope (Eclipse Ni‐U, Nikon) equipped with CCD camera (DS‐Fi1c, Nikon) using the software NIS‐Elements F4.6 (Nikon, RRID:SCR_014329). The image data were analyzed with the software Image‐Pro Plus (Version 6.0, Media Cybernetic, Rockville, MD, RRID:SCR_007369). Fluorescent staining with PKCα (RRID:AB_777294) or PKCβII (RRID:AB_779042) antibody was examined on randomly selected neurons. Fluorescent intensities were measured along a straight line drawn across the observed cells and plotted after subtracting the background baseline fluorescence (see Figures 7 and S3).

### 2.6 Statistical analysis

All data are expressed as mean ± *SD*. Multiple tests for examining normality or variance of data were performed to determine which type of statistic tests should be used. Unpaired or paired *t* test, or Wilcoxon single rank test, Dunnett's, or Bonferroni's post hoc test following one‐way ANOVA was used for the data analysis; *p* < 0.05 was considered statistically significant.

## 3 RESULTS

### 3.1 The regulation of the AP firing activity of ARC neurons by PKAs, PKCs, or SFKs

**Figure 1:**
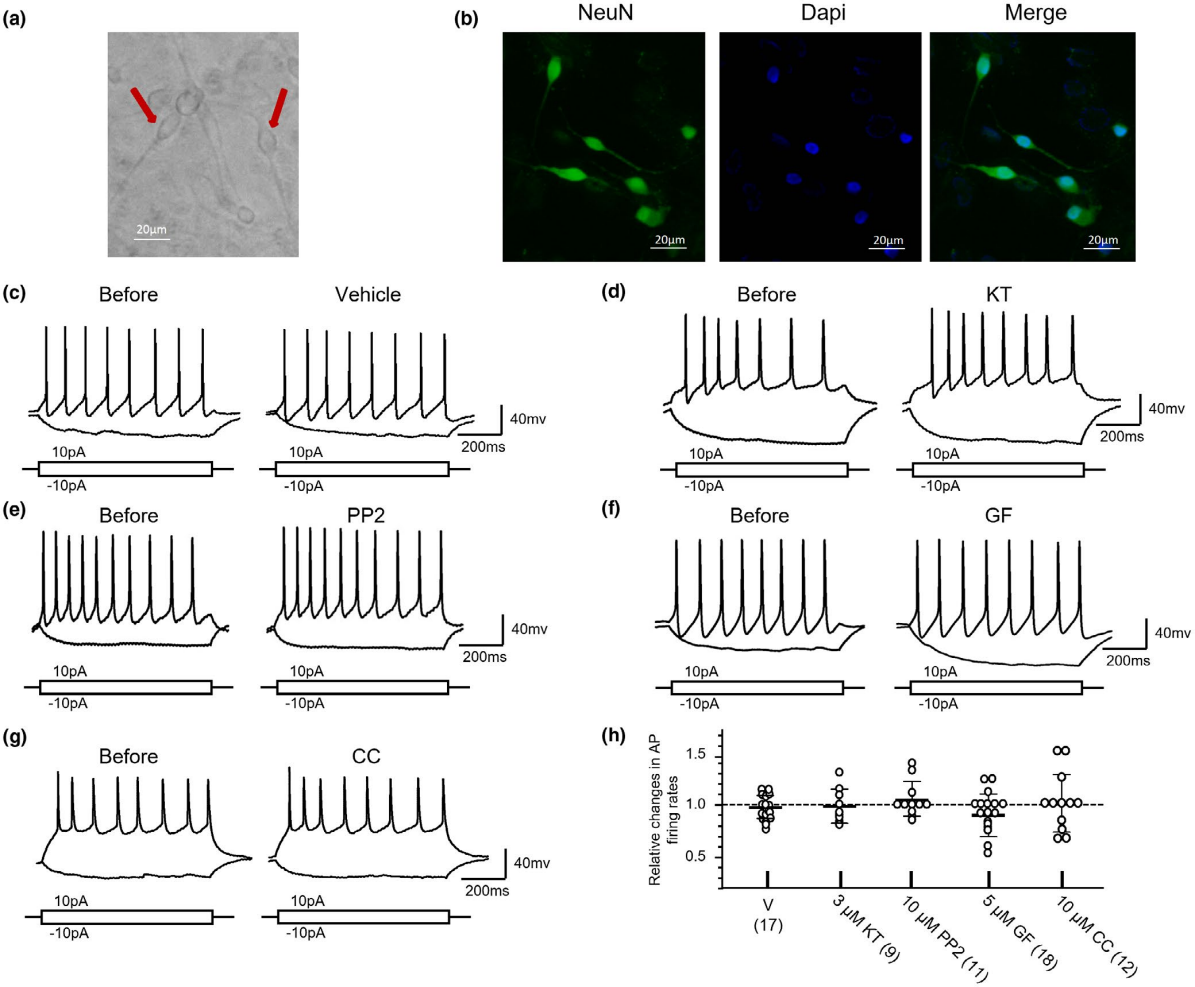
Effects of application of a protein kinase A family (PKA), protein kinase C family (PKC), or Src family kinase (SFK) inhibitor on the action potential (AP) firing activity induced by depolarizing current injections into arcuate nucleus (ARC) neurons. (a) An example of images of ARC neurons in culture; (b) An example of co‐labeling of cultured ARC neurons with NeuN antibody (RRID:AB_2298772; green) and DAPI (blue); (c) Examples of voltage traces recorded before and after bath application of vehicle for 5 min; (d) Examples of voltage traces recorded before and after bath application of KT5720 (KT) (3 μM) for 5 min; (e) Examples of voltage traces recorded before and after bath application of 4‐Amino‐5‐(4‐chlorophenyl)‐7‐(t‐butyl)pyrazolo[3,4‐d]pyrimidine (PP2) (10 μM) for 5 min; (f) Examples of voltage traces recorded before and after bath application of GF109203X (GF) (5 μM) for 5 min; (g) Examples of voltage traces recorded before and after bath application of chelerythrine chloride (CC) (10 μM) for 5 min; Current injection profiles are shown below voltage traces. (h) Summary data (mean ± *SD*) showing AP firing rates relative to those before application of vehicle or the inhibitors (= 1, dashed line). When compared with the effect of vehicle application, no statistically significant difference was found following KT, GF, PP2, or CC application [*p* > 0.05, Bonferroni's post hoc test in comparing with the effect produced by vehicle application, in one‐way ANOVA (*p* = 0.3679, *F*_4,62_ = 1.093)]. Values in brackets indicate the number of ARC neurons tested [Color figure can be viewed at https://www.wileyonlinelibrary.com]

Figure 1a shows examples of ARC cells in culture. In ARC cultures 72.3% ± 7.1% (mean ± *SD*) of cells stained with DAPI were found to be co‐labeled with NeuN staining (Figure 1b). In current clamp recordings depolarizing currents (increasing step amplitude: 10 pA; duration: 1 s; injection interval: 600 ms; see Section 2) were injected into recorded cells to induce APs. All of recorded cells were able to generate multiple APs in response to the current injection (see Figure 1c–g). The resting membrane potentials of recorded cells were −59.3 ± 8.9 mV (mean ± *SD*, *n* = 188). The AP thresholds and firing rates were −33.8 ± 5.8 mV and 9.1 ± 3.8 Hz (mean ± *SD*, *n* = 188). The AP amplitudes, AP half widths and first spike latencies were 83.0 ± 11.8 mV, 5.0 ± 2.1 ms, and 175.2 ± 77.3 ms (mean ± *SD*, *n* = 188). The input resistances were 2.6 ± 1.2 GΩ (mean ± *SD*, *n* = 188).

Previous studies have documented that KT is a potent PKA inhibitor with an IC50 of 3.3 μM (Davies, Reddy, Caivano, & Cohen, 2000; Murray, 2008). Application of 20 nM GF directly to purified PKC proteins leads to a reduction in the activity of PKCs by 50% (Toullec et al., 1991). In cellular experimental models the IC50 of GF may be ranged from 0.1 to 1 μM (Son, Hong, Kim, Firth, & Park, 2011). Although the finding that chelerythrine was a potent inhibitor of PKCs with an IC50 of 0.66 μM (Herbert, Augereau, Gleye, & Maffrand, 1990; Ringvold & Khalil, 2017) has been challenged by a number of studies in which the enzyme activity of PKCs was found not to be affected by application of this compound (Lee et al., 1998; Vieira et al., 2015), it has been demonstrated that CC may inhibit the translocation of PKCs (Chao, Chen, & Cheng, 1998; Siomboing et al., 2001). SFK activity can be inhibited by 50% following 4 nM PP2 application *in vitro* and 0.6–18 μM in cellular experimental models (Hanke et al., 1996; Karni et al., 2003).

In our present study effects of the PKA inhibitor KT, the PKC inhibitor GF or CC, or the SFK inhibitor PP2 on the electrophysiological properties of cultured ARC neurons were investigated. Table 2 shows summary data demonstrating effects of treatment with vehicle or the inhibitors of PKAs, PKCs, or SFKs for 5 min on the electrophysiological properties of cultured ARC neurons recorded. In these neurons the effects of FSK were examined in 16 neurons after the test of vehicle application, and in 19 neurons the effects of PMA were examined after the test of vehicle. When compared with those before vehicle application, no significant change in the electrophysiological properties measured could be noted after vehicle application (see Table 2, Figure 1h). Interestingly, no significant change in the AP firing rate could be found following treatment with the inhibitor of PKAs, PKCs, or SFKs while the input resistance after KT treatment, the AP amplitude, and AP half width after GF treatment, and the AP threshold after CC treatment showed statistically significant changes (see Table 2, Figure 1c–h).

**Table 2:** The electrophysiological properties recorded before and after treatment (mean ± *SD*)

Treatment	RMP (mV)		Firing rate (Hz)		AP amplitude (mV)		AP half‐width (ms)		AP threshold (mV)		First spike latency (ms)		Input resistance (GΩ)	
Vehicle *n* = 17	Pre‐	Post	Pre‐	Post	Pre‐	Post	Pre‐	Post	Pre‐	Post	Pre‐	Post	Pre‐	Post
KT *n* = 9, 3 μM	−56.7 ± 5.4	−57.4 ± 4.9	10.1 ± 3.0	9.7 ± 2.9	84.5 ± 10.6	82.3 ± 9.3	5.2 ± 1.2	5.3 ± 1.3	−31.4 ± 6.6	−32.3 ± 6.1	151.0 ± 28.9	156.6 ± 33.4	2.2 ± 0.9	2.2 ± 0.8
		(1.02 ± 0.07)		(0.97 ± 0.12)		(0.98 ± 0.07)		(1.03 ± 0.17)		(1.04 ± 0.08)		(1.04 ± 0.14)		(1.01 ± 0.12)
	−58.5 ± 8.4	−58.7 ± 8.5	8.8 ± 2.7	8.7 ± 3.2	84.4 ± 15.2	82.5 ± 17.3	4.5 ± 1.4	4.8 ± 2.1	−35.0 ± 5.8	−36.0 ± 4.7	167.6 ± 59.6	169.1 ± 61.0	2.8 ± 1.2	2.6 ± 1.0*
		(1.01 ± 0.10)		(0.98 ± 0.16)		(0.97 ± 0.08)		(1.05 ± 0.11)		(1.04 ± 0.05)		(1.00 ± 0.16)		(0.94 ± 0.07)*
GF *n* = 18, 5 μM	−56.6 ± 8.1	−57.3 ± 9.3	11.7 ± 3.9	10.5 ± 3.8	82.8 ± 10.1	76.5 ± 13.8	4.1 ± 1.3	4.7 ± 1.9*	−32.2 ± 4.3	−33.9 ± 6.0	168.0 ± 73.0	163.6 ± 62.1	3.4 ± 1.5	3.3 ± 1.4
		(1.01 ± 0.08)		(0.91 ± 0.19)		(0.93 ± 0.13)*		(1.1 ± 0.24)*		(1.05 ± 0.12)		(1.00 ± 0.21)		(0.99 ± 0.08)
CC *n* = 12, 10 μM	−61.1 ± 8.0	−63.2 ± 8.8	4.2 ± 1.6	4.2 ± 1.8	91.2 ± 19.2	97.5 ± 14.1	8.0 ± 3.6	7.8 ± 3.7	−32.7 ± 5.9	−34.4 ± 5.6*	269.8 ± 121.9	302.7 ± 157.6	2.4 ± 0.4	2.3 ± 0.4
		(1.04 ± 0.10)		(1.01 ± 0.28)		(1.09 ± 0.16)		(0.99 ± 0.28)		(1.06 ± 0.07)*		(1.22 ± 0.70)		(0.98 ± 0.08)
PP2 *n* = 11, 10 μM	−55.3 ± 4.7	−57.1 ± 6.8	9.8 ± 3.0	10.0 ± 2.4	81.5 ± 6.8	78.0 ± 10.1	5.1 ± 1.8	5.2 ± 1.6	−30.2 ± 5.3^	−32.2 ± 5.4	149.1 ± 76.7^	143.2 ± 34.9	2.8 ± 0.7	2.9 ± 0.7
		(1.03 ± 0.12)		(1.05 ± 0.17)		(0.96 ± 0.10)		(1.05 ± 0.17)		(1.08 ± 0.20)		(1.04 ± 0.23)		(1.03 ± 0.07)
PP3 *n* = 8, 10 μM	−65.0 ± 9.1	−64.7 ± 10.2	7.6 ± 3.0	7.5 ± 3.1	75.3 ± 13.1	73.6 ± 16.2	4.1 ± 0.6	4.4 ± 0.6	−43.2 ± 4.4	−42.1 ± 4.7	153.9 ± 38.0^	158.4 ± 48.0	2.0 ± 0.8	1.98 ± 0.8
		(1.00 ± 0.06)		(0.99 ± 0.14)		(0.97 ± 0.07)		(1.07 ± 0.09)		(0.97 ± 0.04)		(1.02 ± 0.10)		(1.00 ± 0.09)
FSK *n* = 15, 1 μM	−59.1 ± 7.5	−60.9 ± 6.7	9.3 ± 3.0	9.7 ± 3.1	81.6 ± 11.9	80.2 ± 11.5	6.0 ± 2.5^	5.9 ± 2.2	−32.2 ± 3.5	−33.3 ± 3.6	148.9 ± 35.9	150.9 ± 27.6	3.0 ± 1.1	3.0 ± 1.2
		(1.04 ± 0.10)		(1.06 ± 0.15)		(0.99 ± 0.11)		(1.02 ± 0.14)		(1.04 ± 0.07)		(1.06 ± 0.26)		(1.03 ± 0.11)
FSK *n* = 13, 10 μM	−59.3 ± 4.0	−57.2 ± 5.7	9.9 ± 2.9	11.5 ± 3.6***	84.9 ± 10.2	80.7 ± 11.0	5.0 ± 2.6^	4.9 ± 1.9	−30.3 ± 2.9	−31.0 ± 4.5	159.2 ± 28.9	148.0 ± 36.6	2.8 ± 1.2	2.9 ± 1.1
		(0.96 ± 0.06)		(1.18 ± 0.12)***		(0.95 ± 0.09)		(1.03 ± 0.12)		(1.02 ± 0.09)		(0.93 ± 0.16)		(1.05 ± 0.11)
FSK *n* = 15, 50 μM	−58.6 ± 12.4^	−57.2 ± 9.1	11.1 ± 4.7	13.5 ± 4.4***	80.7 ± 14.9	75.8 ± 11.4	4.4 ± 1.9^	4.8 ± 1.8	−33.6 ± 5.0	−35.0 ± 5.8	167.2 ± 90.6^	133.3 ± 60.4	1.9 ± 1.0	1.9 ± 0.9
		(0.99 ± 0.09)		(1.29 ± 0.21)***		(0.95 ± 0.14)		(1.11 ± 0.25)		(1.04 ± 0.08)		(0.84 ± 0.21)		(0.84 ± 0.21)*
FSK *n* = 11, 100 μM	−58.2 ± 4.2	−55.3 ± 6.6	10.6 ± 3.3	14.3 ± 3.0***	86.1 ± 8.3	83.0 ± 9.2	4.3 ± 1.8^	4.2 ± 1.3	−30.4 ± 5.0	−28.3 ± 10.1	157.1 ± 32.9	132.6 ± 17.0*	2.8 ± 1.0	2.8 ± 0.9
		(0.95 ± 0.13)		(1.42 ± 0.32)**		(0.97 ± 0.10)		(0.99 ± 0.17)		(0.92 ± 0.28)		(0.87 ± 0.16)*		(1.01 ± 0.11)
PMA *n* = 19, 0.1, μM	−59.3 ± 7.2	−62.5 ± 11.4	7.8 ± 2.5	9.2 ± 2.3**	83.7 ± 8.9	75.8 ± 8.6***	6.4 ± 1.8^	6.8 ± 1.8	−33.4 ± 5.5	−39.5 ± 12.3*	147.6 ± 35.2	143.0 ± 39.0	3.3 ± 1.4	3.4 ± 1.3
		(1.06 ± 0.16)		(1.22 ± 0.31)**		(0.91 ± 0.11)**		(1.10 ± 0.23)*		(1.19 ± 0.35)		(1.10 ± 0.23)		(1.03 ± 0.11)
PMA *n* = 19, 1 μM	−58.5 ± 6.5^	−57.8 ± 9.4	8.8 ± 4.2	10.8 ± 4.4***	78.9 ± 9.0	68.7 ± 8.8***	5.3 ± 1.6	6.0 ± 1.7	−34.4 ± 6.1	−39.1 ± 10.1*	156.4 ± 48.5	132.1 ± 35.7**	2.4 ± 1.3^	2.5 ± 1.4
		(0.99 ± 0.15)		(1.28 ± 0.29)***		(0.88 ± 0.12)***		(1.14 ± 0.26)*		(1.16 ± 0.38)**		(0.86 ± 0.17)*		(1.02 ± 0.10)
PMA *n* = 15, 5 μM	−59.7 ± 10.0	−55.5 ± 8.8**	7.3 ± 2.8	9.6 ± 3.5**	85.9 ± 11.7	76.1 ± 13.7***	5.0 ± 1.6	5.6 ± 2.1*	−33.0 ± 4.2	−34.2 ± 6.1	219.7 ± 108.8^	191.0 ± 78.4	2.8 ± 1.0	2.6 ± 0.9*
		(0.93 ± 0.08)**		(1.37 ± 0.39)**		(0.88 ± 0.08)**		(1.11 ± 0.16)*		(1.03 ± 0.11)		(0.93 ± 0.23)		(0.95 ± 0.08)*
PMA *n* = 16, 10 μM	−63.0 ± 15.3	−61.2 ± 12.9	9.1 ± 4.7^	11.6 ± 5.3***	85.8 ± 11.7	78.8 ± 11.6	3.7 ± 1.4	4.2 ± 2.0	−37.1 ± 7.5	−37.7 ± 6.9	163.3 ± 62.5^	166.0 ± 67.2	2.3 ± 1.1^	2.2 ± 1.1
		(0.99 ± 0.17)		(1.34 ± 0.26)***		(0.93 ± 0.18)		(1.10 ± 0.23)		(1.02 ± 0.11)		(0.92 ± 0.41)		(0.97 ± 0.09)
EPQ(pY)EEIPIA *n* = 13, 1 mM	−63.7 ± 7.1	−59.1 ± 5.0*	7.3 ± 3.0^	9.9 ± 3.0**	87.2 ± 10.3	76.1 ± 10.9***	3.4 ± 1.0	3.7 ± 1.1	−35.8 ± 6.1^	−37.0 ± 5.3	188.9 ± 49.7	151.9 ± 40.2*	1.8 ± 0.9	1.8 ± 0.8
		(0.94 ± 0.11)		(1.45 ± 0.41)**		(0.87 ± 0.09)***		(1.07 ± 0.16)		(1.04 ± 0.06)*		(0.84 ± 0.22)*		(0.98 ± 0.08)
EPQYEEIPIA	−57.7 ± 6.2	−58.9 ± 5.6	11.0 ± 3.1	10.9 ± 3.0	79.3 ± 11.5	79.9 ± 10.1	4.6 ± 1.6^	4.4 ± 1.4	−32.9 ± 6.5^	−34.3 ± 6.1	148.57 ± 39.2^	146.79 ± 30.7	1.9 ± 0.8	2.0 ± 0.9*
*n* = 12, 1 mM		(1.02 ± 0.06)		(1.00 ± 0.09)		(1.01 ± 0.07)		(0.98 ± 0.13)		(1.05 ± 0.11)		(1.01 ± 0.11)		(1.05 ± 0.07)*

Note

Summary data showing changes in the electrophysiological properties of 188 neurons (which did not include those neurons pre‐treated with antagonists of PKAs, PKCs, or SFKs) following treatment as indicated for 5 min are presented. In these neurons, the effects of FSK were examined in 16 neurons after the test of vehicle application, and in 19 neurons the effects of PMA were examined after the test of vehicle. The input resistance of whole cells was determined by Δ*V*/*I*_injection_ (Δ*V*: changes in the steady state membrane potential in response to the injection of −10 pA currents; *I*_injection_: currents (−10 pA) injected into neurons. With the exception of those data indicated with ^ (*p* < 0.05, KS normality test), all the data of the resting membrane potential (RMP), firing rate, action potential (AP) amplitudes, AP half width, AP threshold and first spike of latency, and input resistance of ARC neurons in each group before treatment passed normality test (*p* > 0.05, KS normality test). Relative values to those before treatment (= 1) are shown in brackets. FSK was co‐applied with IBMX (50 μM). The data following intracellular application of EPQ(pY)EEIPIA or EPQYEEIPIA shown in this table were recorded at “0” min (pre‐, immediately after breakthrough) and 10 min after breakthrough. *n*: number of neurons tested. *, **, ***: *p* < 0.05, 0.01, 0.001 in paired *t* test in comparisons of the absolute or relative values with those before treatments or in Wilcoxon single rank test in comparisons of the absolute values with that before treatment with 10 μM PMA or 1 mM EPQ(pY)EEIPIA.

**Figure 2:**
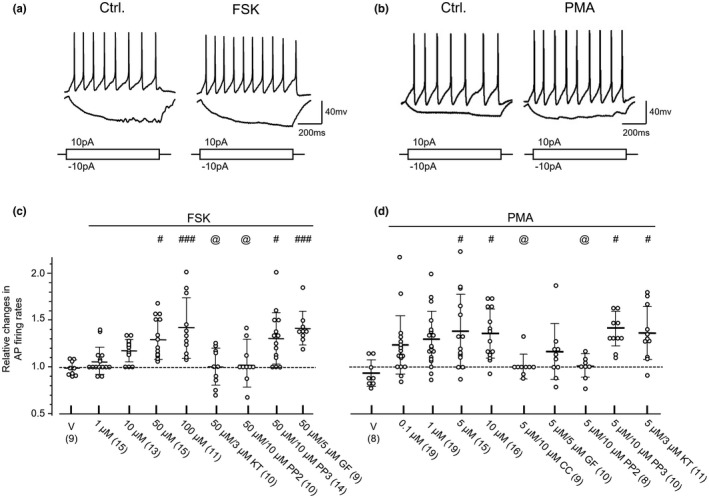
Effects of application of a protein kinase A family (PKA) or protein kinase C family (PKC) activator on the action potential (AP) firing activity induced by depolarizing current injections into arcuate nucleus (ARC) neurons. (a) Examples of voltage traces recorded before and after bath application of forskolin (FSK) (50 μM) co‐applied with 3‐Isobutyl‐1‐methylxanthine (IBMX) (50 μM) for 5 min; (b) Examples of voltage traces recorded before and after bath application of Phorbol 12‐myristate 13‐acetate (PMA) (5 μM) for 5 min; Current injection profiles are shown below voltage traces. (c) Summary data (mean ± *SD*) showing AP firing rates relative to those before the bath application (= 1, dashed lines) of FSK; V: vehicle application; doses of FSK applied are shown underneath each column. 50 μM/3 μM KT: 50 μM FSK were applied to neurons pre‐treated with KT5720 (KT) (3 μM) for 30 min; 50 μM/10 μM PP2: 50 μM FSK were applied to neurons pre‐treated with 4‐Amino‐5‐(4‐chlorophenyl)‐7‐(t‐butyl)pyrazolo[3,4‐d]pyrimidine (PP2) (10 μM) for 30 min; 50 μM/10 μM 4‐Amino‐7‐phenylpyrazol[3,4‐d]pyrimidine (PP3): 50 μM FSK were applied to neurons pre‐treated with PP3 (10 μM) for 30 min; #, ###: *p* < 0.05, 0.001, Bonferroni's post hoc test in comparing with the effects produced by vehicle application, following one‐way ANOVA (*p* < 0.0001, *F*_8,97_ = 6.45). @: *p* < 0.05, Bonferroni's post hoc test in comparisons between the effect produced by application of 50 μM FSK to neurons pre‐treated with KT, PP2, PP3, or GF109203X (GF) versus that to neurons without pre‐treatment, following one‐way ANOVA. (d) Summary data (mean ± *SD*) showing AP firing rates relative to those before bath application (= 1, dashed lines) of PMA; V: vehicle application; doses of PMA applied are shown underneath each column. 5 μM/10 μM chelerythrine chloride (CC): 5 μM PMA were applied to neurons pre‐treated with CC (10 μM) for 30 min; 5 μM/5 μM GF: 5 μM PMA were applied to neurons pre‐treated with GF (5 μM) for 30 min; 5 μM/10 μM PP2: 5 μM PMA were applied to neurons pre‐treated with PP2 (10 μM) for 30 min; 5 μM/10 μM PP3: 5 μM PMA were applied to neurons pre‐treated with PP3 (10 μM) for 30 min; #: *p* < 0.05, Bonferroni's post hoc test in comparing with the effect produced by vehicle application following one‐way ANOVA (*p* < 0.0004, *F*_9,115_ = 3.76). @: *p* < 0.05, Bonferroni's post hoc test in comparisons between the effects produced by application of 5 μM PMA to neurons pre‐treated with CC, GF, PP2, PP3, or KT versus that to neurons without pre‐treatment, in one‐way ANOVA

**Figure 3:**
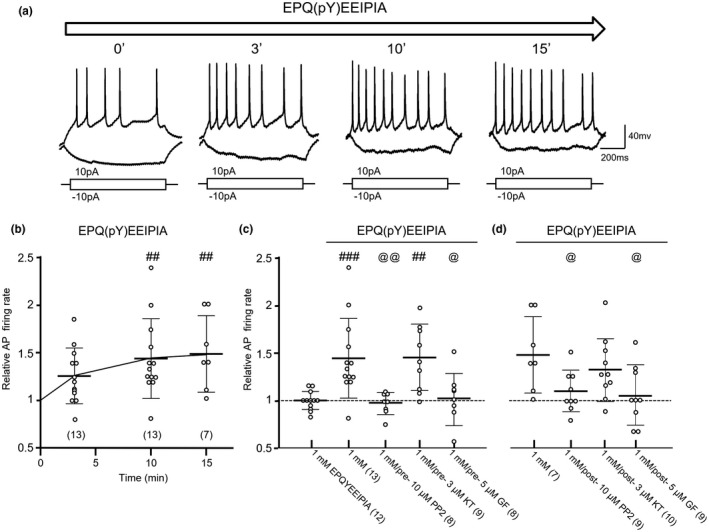
Effects of an Src family kinase (SFK) activation peptide applied into arcuate nucleus (ARC) neurons on the action potential (AP) firing activity induced by depolarizing current injections. (a) Examples of voltage traces recorded from an ARC neuron with an electrode filled with the internal solution containing the Src family kinase (SFK) activation peptide EPQ(pY)EEIPIA (1 mM) immediately (“0” min) and 3, 10, and 15 min after breakthrough. (b) Summary data (mean ± *SD*) showing AP firing rates relative to those recorded at “0” min (= 1). ##: *p* < 0.01, Dunnett's post hoc test in comparisons with those recorded at “0” min, following one‐way ANOVA (*p* < 0.0056, *F*_3,39_ = 4.884). (c) Summary data (mean ± *SD*) showing effects of intracellularly delivered EPQYEEIPIA (1 mM) at 10 min after breakthrough and the effects of 4‐Amino‐5‐(4‐chlorophenyl)‐7‐(t‐butyl)pyrazolo[3,4‐d]pyrimidine (PP2) (10 μM), KT5720 (KT) (3 μM), or GF109203X (GF) (5 μM), each of them was bath applied to neurons for 30 min before EPQ(pY)EEIPIA was delivered into the neurons through breakthrough. ##, ###: *p* < 0.01, 0.001, Bonferroni's post hoc test following one‐way ANOVA (*p* < 0.0001, *F*_4,45_ = 7.735) in comparing the effect produced by the peptide EPQYEEIPIA application with that produced by the peptide EPQ(pY)EEIPIA application in neurons with or without PP2, KT or GF pre‐treatment for 30 min. @, @@: *p* < 0.05, 0.01; Bonferroni's post hoc test following one‐way ANOVA in comparisons between the effects produced by application of EPQ(pY)EEIPIA in neurons pre‐treated with PP2, KT, or GF and that in neurons without the pre‐treatments. (d) Summary data (mean ± *SD*) showing effects of intracellularly delivered EPQ(pY)EEIPIA (1 mM) at 15 min after breakthrough and the effects of PP2 (post‐, 10 μM), KT (post‐, 3 μM), or GF (post‐, 5 μM), each of them was bath applied to neurons (for 5 min) 10 min after EPQ(pY)EEIPIA was delivered into the neurons through breakthrough. @: *p* < 0.05; Bonferroni's post hoc test following one‐way ANOVA (*p* = 0.027, *F*_3,31_ = 3.498) in comparisons between the effects produced by application of EPQ(pY)EEIPIA alone at 15 min after breakthrough and the effects after PP2 (post‐, 10 μM), KT (post‐, 3 μM), or GF (post‐, 5 μM) was applied to neurons 10 min after EPQ(pY)EEIPIA was delivered into the neurons through breakthrough

We then examined the effects of agents which may enhance the activity of PKAs, PKCs, or SFKs (see Figures 2 and 3). It is known that 5–10 μM FSK may selectively activate the adenylate cyclase by 50% and thereby enhance PKA activity (Litosch, Hudson, Mills, Li, & Fain, 1982; Seamon, Padgett, & Daly, 1981). To examine the effect of PKA activation, in this work FSK at the concentration of 1, 10, 50, or 100 µM was co‐applied with 50 μM of IBMX, a non‐competitive inhibitor of phosphodiesterase (IC50: 50 μM) (Essayan, 2001) (Table 2, Figure 2a). A dose‐dependent increase was noted in the AP firing rate following FSK application when compared with those before the application of FSK (Table 2, Figure 2c). In comparison with the effect of vehicle application, the AP firing rate was significantly enhanced when 50 or 100 μM of FSK was bath applied (*p* < 0.05, Bonferroni's post hoc test in one‐way ANOVA [*p* < 0.0001, *F*_4,58_ = 8.712]; see Figure 2). In comparing with the effect of application of vehicle or FSK (50 µM) alone the increase in the AP firing activity induced by 50 µM FSK was blocked in neurons pre‐treated with the PKA inhibitor KT (3 μM) or the SFK inhibitor PP2 (10 μM) for 30 min. Pre‐treatment with the PKC inhibitor GF (5 μM) or PP3 (10 μM, an inactive form of PP2) had no such effect (see Figure 2c). These data implicate that SFKs may be involved in the regulation of the AP firing activity of ARC neurons by PKAs.

Previous studies have documented that PKC activity can be enhanced by 50% following the application of 0.1–0.2 μM PMA *in vitro* and in cellular models (Bozou, Rochet, Magnaldo, Vincent, & Kitabgi, 1989; Niedel, Kuhn, & Vandenbark, 1983). In this work a dose‐dependent increase was also noted in the AP firing rate following PMA application (see Table 2 and Figure 2d). When compared with that induced by vehicle application, we found that bath application of PMA (≥5 μM) significantly increased the AP firing activity (*p* < 0.05, Bonferroni's post hoc test in one‐way ANOVA [*p* = 0.02, *F*_4,72_ = 3.129]; Figure 2d). We also found that after the application of 5 μM PMA the resting membrane potentials (RMPs) were reduced from −59.7 ± 10.0 to −55.5 ± 8.8 mV (mean ± *SD*, *n* = 15), which was statistically significant (*p* = 0.004, *t*_14_ = 3.48, paired *t* test, see Table 2). With the exception of application of 10 μM PMA the AP amplitude was significantly reduced following treatments with PMA (see Table 2). Significant increases in the AP half width and decreases in the AP threshold were found in neurons treated with PMA at concentrations of 0.1–5 μM and 0.1–1 μM, respectively (see Table 2).

In comparing with the effect of application of vehicle or PMA (5 µM) alone the increase in the AP firing activity induced by 5 µM PMA was blocked in neurons pre‐treated with the PKC inhibitor CC (10 μM) or GF (5 μM), or the SFK inhibitor PP2 (10 μM) for 30 min. Pre‐treatment with the PKA inhibitor KT (3 μM) or PP3 (10 μM, an inactive form of PP2) had no such effect (see Figure 2d). These data implicate that SFKs may also be involved in the regulation of the AP firing activity of ARC neurons by PKCs.

Previous studies have shown that the peptide EPQ(pY)EEIPIA may bind to the SH2 domain of SFKs and thereby activate the kinases (Liu et al., 1993; Xu, Doshi, Lei, Eck, & Harrison, 1999). Delivering 1 mM of the peptide into cultured neurons (Yu, Askalan, Keil, & Salter, 1997) or 5 mM of the peptide into hippocampal neurons in brain slice preparations (Chichorro, Porreca, & Sessle, 2017) significantly enhances SFK‐mediated neuronal activity. In this work, we investigated effects of the peptide EPQ(pY)EEIPIA delivered into neurons on the firing activity. Since the peptide EPQ(pY)EEIPIA is not membrane‐permeant (Liu et al., 1993; Xu et al., 1999), to examine the effect of direct activation of SFKs, the peptide was delivered into ARC neurons through recording electrodes filled with the internal solution containing the EPQ(pY)EEIPIA (1 mM) (Chichorro et al., 2017; Feng et al., 2012; Yu et al., 1997).

Figure 3a shows examples of current traces recorded from an ARC neuron at “0” (immediately), 3, 10, and 15 min after breakthrough with electrodes filled with the internal solution containing the EPQ(pY)EEIPIA (1 mM). When compared with those recorded immediately after breakthrough (at “0” min) the AP firing rate and AP threshold recorded at 10 min after breakthrough was significantly increased while the RMP, AP amplitude, and first spike latency were decreased (see Table 2, Figure 3). In contrast, the intracellular application of the non‐phosphorylated peptide EPQYEEIPIA (1 mM) produced no such effects but increased the input resistance of neurons recorded (see Table 2 and Figure 3c). In comparing with the effect of application of EPQYEEIPIA (1 mM) or EPQ(pY)EEIPIA (1 mM) alone the increase in the AP firing activity induced by 1 mM EPQ(pY)EEIPIA was blocked in neurons pre‐treated with the PKC inhibitor GF (5 μM), or the SFK inhibitor PP2 (10 μM) for 30 min. Pre‐treatment with the PKA inhibitor KT (3 μM) had no such effect (see Figure 3c).

Furthermore, we found that the effect of EPQ(pY)EEIPIA application could also be reversed by bath applications of GF (5 μM) or PP2 (10 μM) to neurons at 10 min after EPQ(pY)EEIPIA was delivered through breakthrough (Figure 3d). The PKA inhibitor KT (3 μM) had no such effect (Figure 3d). Thus, PKAs could up‐regulate the AP firing activity of ARC neurons via SFKs while PKCs and SFKs interact reciprocally to regulate the AP firing activity.

### 3.2 Phosphorylation regulation of PKAs, PKCs, or SFKs in cultured ARC cells

**Figure 4:**
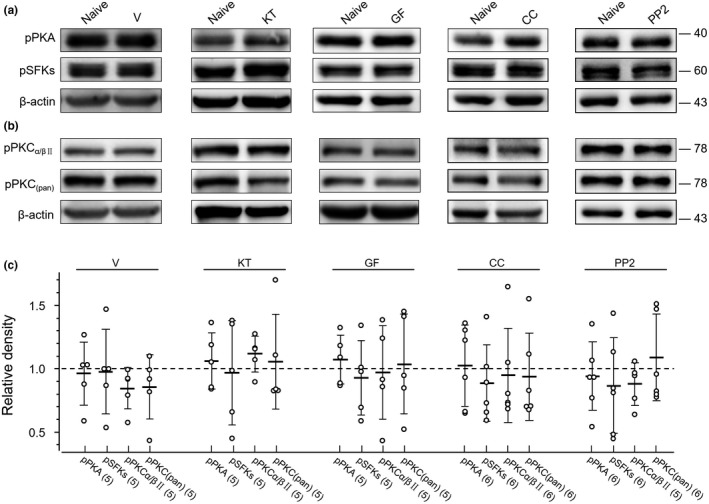
Effects of application of a protein kinase A family (PKA), protein kinase C family (PKC), or Src family kinase (SFK) inhibitor on the phosphorylation of PKAs, PKCs, or SFKs. (a) The gels loaded with lysates prepared from cultured arcuate nucleus (ARC) cells without any treatment (naïve) or treated for 30 min with vehicle (V), KT5720 (KT) (3 μM), GF (5 μM), chelerythrine chloride (CC) (10 μM), or 4‐Amino‐5‐(4‐chlorophenyl)‐7‐(t‐butyl)pyrazolo[3,4‐d]pyrimidine (PP2) (10 μM) as indicated. Each group of blots was cropped from the same PVDF membrane, stripped and successively probed with antibodies of pPKAs (RRID:AB_1524202), pSFKs (RRID:AB_10860257), and β‐actin (RRID:AB_2687938) as indicated on the left of blots. (b) The gels loaded with lysates prepared from cultured ARC cells without any treatment (naïve) or treated for 30 min with vehicle (V), KT5720 (KT) (3 μM), GF109203X (GF) (5 μM), CC (10 μM), or PP2 (10 μM) as indicated. Each group of blots was cropped from the same PVDF membrane, stripped, and successively probed with antibodies of pPKCα/βII (RRID:AB_2284224), pPKCpan (RRID:AB_2168219), and β‐actin (RRID:AB_2687938) as indicated on the left of blots. Values on the right side of blots indicate the molecular mass (Kd). The ratio of band intensities versus that of β‐actin was normalized to the ratio in naïve cells (= 1, dashed line) for determining relative changes. (c) Summary data (mean ± *SD*) of the relative changes. Values in brackets indicate the number of experimental repeats

The phosphorylation of PKAs at the residue T197 (Montenegro, Masgrau, Gonzalez‐Lafont, Lluch, & Garcia‐Viloca, 2012; Seifert et al., 2002), PKCs at the residues equivalent to S660 of PKCβII or PKCα/βII at T638/641 (Antal, Callender, Kornev, Taylor, & Newton, 2015; Freeley, Kelleher, & Long, 2011; Keranen, Dutil, & Newton, 1995) or SFKs at the residues equivalent to Y416 of chicken c‐Src (Groveman et al., 2012; Salter & Kalia, 2004; Thomas & Brugge, 1997; Yu & Groveman, 2012) has been found to be related to the functional status of these kinases. Furthermore, we investigated the phosphorylation of these kinases in ARC cells to understand mechanisms which may underlie the regulation of the AP firing activity in cultured ARC neurons by PKAs, PKCs, or SFKs. We used an antibody (RRID:AB_1524202) which recognizes phosphorylated T197 in PKAs (pPKAs), a phospho‐PKC antibody (RRID:AB_2168219; pan, pPKCs) which detects the phosphorylation of PKCα, βI, βII, δ, ε, and η isoforms at the sites equivalent to S660 of PKCβII (see manufacturer's documents at https://www.cellsignal.com), and an antibody (RRID:AB_2284224) which detects the phosphorylated T638/641 of PKCα/βII (pPKCα/βII), and a phospho‐SFK (pSFKs) antibody (RRID:AB_10860257) which recognizes several member of this family (such as Src, Fyn, Yes, and Lyn) phosphorylated at the sites equivalent to Y416 in c‐Src (see manufacturer's documents at https://www.cellsignal.com) (see Table 1). Compared with those in naïve cells treated only with culture medium, no significant change in the expression of pPKAs (*p* = 0.61, *t*_8_ = 0.52; unpaired *t* test), pPKCs (*p* = 0.26, *t*_8_ = 1.2; unpaired *t* test), pPKCα/βII (*p* = 0.08, *t*_8_ = 2.03; unpaired *t* test) and pSFKs (*p* = 0.89, *t*_8_ = 0.15; unpaired *t* test) in cells treated with vehicle for 30 min (see Figure 4). Similarly, no significant change in the expression of pPKCs, pPKAs, and pSFKs was found in cells treated with KT (3 μM), GF (5 μM), CC (10 μM), or PP2 (10 μM) for 30 min when compared with those in naïve cells (see Figure 4).

**Figure 5:**
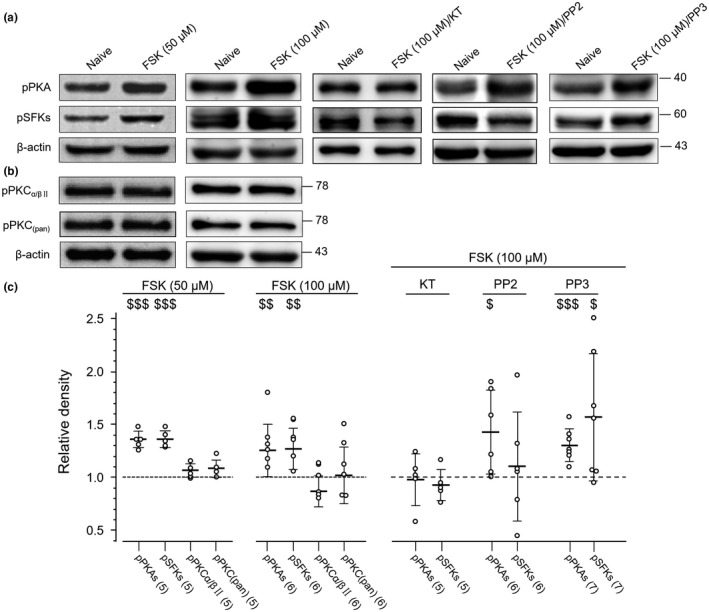
Effects of application of the adenylate cyclase activator forskolin (FSK) on the phosphorylation of protein kinase A family (PKAs), protein kinase C family (PKCs), or Src family kinase (SFKs) in cultured arcuate nucleus (ARC) cells. (a) The gels were loaded with lysates prepared from cultured ARC cells without any treatment (naïve) or cells which were treated only with 50 or 100 μM FSK, or treated with KT5720 (KT) (3 μM,/KT), 4‐Amino‐5‐(4‐chlorophenyl)‐7‐(t‐butyl)pyrazolo[3,4‐d]pyrimidine (PP2) (10 μΜ,/PP2), or 4‐Amino‐7‐phenylpyrazol[3,4‐d]pyrimidine (PP3) (10 μΜ,/PP3) for 30 min before application of 100 μM FSK. Each group of blots was cropped from the same PVDF membrane, stripped, and successively probed with antibodies of pPKAs (RRID:AB_1524202), pSFKs (RRID:AB_10860257), and β‐actin (RRID:AB_2687938) as indicated on the left of blots. (b) The gels were loaded with lysates prepared from cultured ARC cells without any treatment (naïve) or cells which were treated only with 50 or 100 μM FSK. Each group of blots was cropped from the same PVDF membrane, stripped, and successively probed with pPKCα/βII (RRID:AB_2284224), pPKCpan (RRID:AB_2168219), and β‐actin (RRID:AB_2687938) antibodies as indicated on the left of blots. Examples of the blots from the same full length PVDF membranes were shown in Figure S2. The ratio of band intensities versus that of β‐actin was normalized to the ratio in naïve cells (= 1, dashed line) for determining relative changes. Values on the right side of blots indicate the molecular mass (Kd). (c) Summary data (mean ± *SD*) of the relative changes in pPKAs, pSFKs, pPKCα/βII, and pPKCpan following treatment with 50 or 100 μΜ FSK. $, $$, $$$: *p* < 0.05, 0.01, 0.001, unpaired *t* test in comparing with that in naïve cells (= 1, dashed line). Values in brackets indicate the number of experimental repeats

We then examined effects of bath application of FSK or PMA on the phosphorylation of PKAs, PKCs, or SFKs in cultured ARC cells. When compared with that in naïve cells, a significant increase was found in the expression of pPKAs (*p* = .008, *t*_10_ = 3.31, unpaired *t* test) or pSFKs (*p* = 0.001, *t*_10_ = 4.4, unpaired *t* test) following treatment with FSK (50 μM) for 30 min (see Figure 5). No statistically significant change was noted in the expression of the protein PKAs (*p* = 0.34, *t*_10_ = 1.01, unpaired *t* test) or Src (*p* = 0.11, *t*_10_ = 1.75, unpaired *t* test) when compared with that in naïve cells (see Figure S4a). When compared with that in naïve cells, the ratio of the phosphorylated versus total PKAs was increased by 20% ± 17% (mean ± *SD*, *p* = 0.018, *t*_10_ = 2.8, unpaired *t* test) (see Figure S4a).

When compared with those in naïve cells, the FSK application did not induce any significant change in pPKCs (*p* = 0.39, *t*_10_ = 0.90, unpaired *t* test) or pPKCα/βII (*p* = 0.47, *t*_10_ = 0.75, unpaired *t* test) (see Figure 5). In cells pre‐treated with KT (3 μM for 30 min) the FSK application did not induce any significant increase in the expression of pPKAs or pSFKs (see Figure 5). In cells pre‐treated with either PP3 (10 μM) or PP2 (10 μM) for 30 min the FSK application still induced significant increases in the expression of pPKAs (PP2: *p* = 0.02, *t*_10_ = 2.64; PP3: *p* = 0.0002, *t*_12_ = 5.22, unpaired *t* test; see Figure 5). However, no increase in pSFKs was induced by FSK application in cells pre‐treated with PP2 (*p* = 0.63, *t*_10_ = 0.5, unpaired *t* test; see Figure 5). In cells pre‐treated with PP3 (10 μM) the FSK application induced a significant increase in the expression of pSFKs (*p* = 0.011, *t*_11_ = 2.05, unpaired *t* test; see Figure 5).

**Figure 6:**
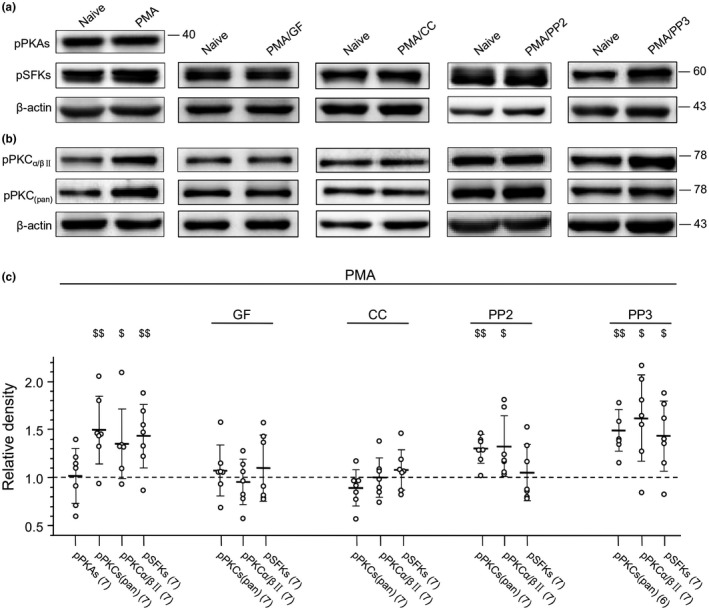
Effects of application of the protein kinase C family (PKC) activator Phorbol 12‐myristate 13‐acetate (PMA) on the phosphorylation of PKAs, PKCs or SFKs in cultured arcuate nucleus (ARC) cells. (a) The gels were loaded with lysates prepared from cultured ARC cells without any treatment (naïve) or cells which were treated only with PMA (10 μM) or treated with GF (5 μM,/GF), CC (10 μΜ,/CC), 4‐Amino‐5‐(4‐chlorophenyl)‐7‐(t‐butyl)pyrazolo[3,4‐d]pyrimidine (PP2) (10 μΜ,/PP2) or 4‐Amino‐7‐phenylpyrazol[3,4‐d]pyrimidine (PP3) (10 μΜ,/PP3) for 30 min before application of 10 μM PMA. Each group of blots was cropped from the same PVDF membrane, stripped and successively probed with antibodies as indicated on the left of blots. (b) The gels were loaded with lysates prepared from cultured ARC cells without any treatment (naïve) or cells which were treated only with PMA (10 μM) or treated with GF (5 μM,/GF), CC (10 μΜ,/CC), PP2 (10 μΜ,/PP2), or PP3 (10 μM,/PP3) for 30 min before application of 10 μM PMA. Each group of blots was cropped from the same PVDF membrane, stripped and successively probed with antibodies as indicated on the left of blots. Values on the right side of blots indicate the molecular mass (Kd). (c) Summary data (mean ± *SD*) of the relative changes. $, $$: *p* < 0.05, 0.01, unpaired *t* test in comparison with that in naïve cells (= 1, dashed line). Values in brackets indicate the number of experimental repeats

When compared to that found in naïve cells, we found that PMA (10 μM for 30 min) significantly increased the expression of both pPKCs and pPKCα/βII (pPKCs: *p* = 0.003, *t*_12_ = 3.7; pPKCα/βII: *p* = 0.028, *t*_12_ = 2.5, unpaired *t* test; see Figure 6). While the expression of pPKAs was not affected by the PMA application (*p* = 0.92, *t*_12_ = 0.10, unpaired *t* test), the expression of pSFKs increased significantly (*p* = 0.005, *t*_12_ = 3.42, unpaired *t* test, see Figure 6). When compared to that in naïve cells, no statistically significant change was noted in the expression of protein PKCα (*p* = 0.19, *t*_12_ = 1.4, unpaired *t* test), PKCβII (*p* = 0.051, *t*_12_ = 2.17, unpaired *t* test) or Src (*p* = 0.072, *t*_12_ = 2.0, unpaired *t* test) in cells treated with PMA (10 μM, see Figure S4b).

When compared to that in naïve cells, no significant increase in the expression of pPKCs (GF: *p* = 0.50, *t*_12_ = 0.70; CC: *p* = 0.14, *t*_12_ = 1.6, unpaired *t* test), pPKCα/βII (GF: *p* = 0.57, *t*_12_ = 0.58; CC: *p* = 0.98, *t*_12_ = 0.03, unpaired *t* test) or pSFKs (GF: *p* = 0.48, *t*_12_ = 0.72; CC: *p* = 0.36, *t*_12_ = 0.94, unpaired *t* test) was found following the application of 10 μM PMA for 30 min in cells pre‐treated with GF (5 μM) or CC (10 μM) for 30 min (see Figure 6). In cells pre‐treated with PP2 (10 μM) for 30 min the effect of PMA on the expression of pSFKs was prevented (*p* = 0.67, *t*_12_ = 0.43, unpaired *t* test) while no change was found in the PMA application‐induced increase in the expression of pPKCs or pPKCα/βII (Figure 6). Pre‐treatment with PP3 (10 μM) for 30 min produced no such effects (Figure 6). Since the peptide EPQ(pY)EEIPIA is not membrane‐permeant (Liu et al., 1993; Xu et al., 1999), effects of the direct activation of SFKs on PKAs or PKCs still need to be clarified. Despite this limitation, our data have identified that the activation of PKAs or PKCs may enhance the activity of SFKs and thereby up‐regulate the AP firing activity in ARC neurons.

### 3.3 The regulation of PKCα and PKCβII distributions in ARC neurons

**Figure 7:**
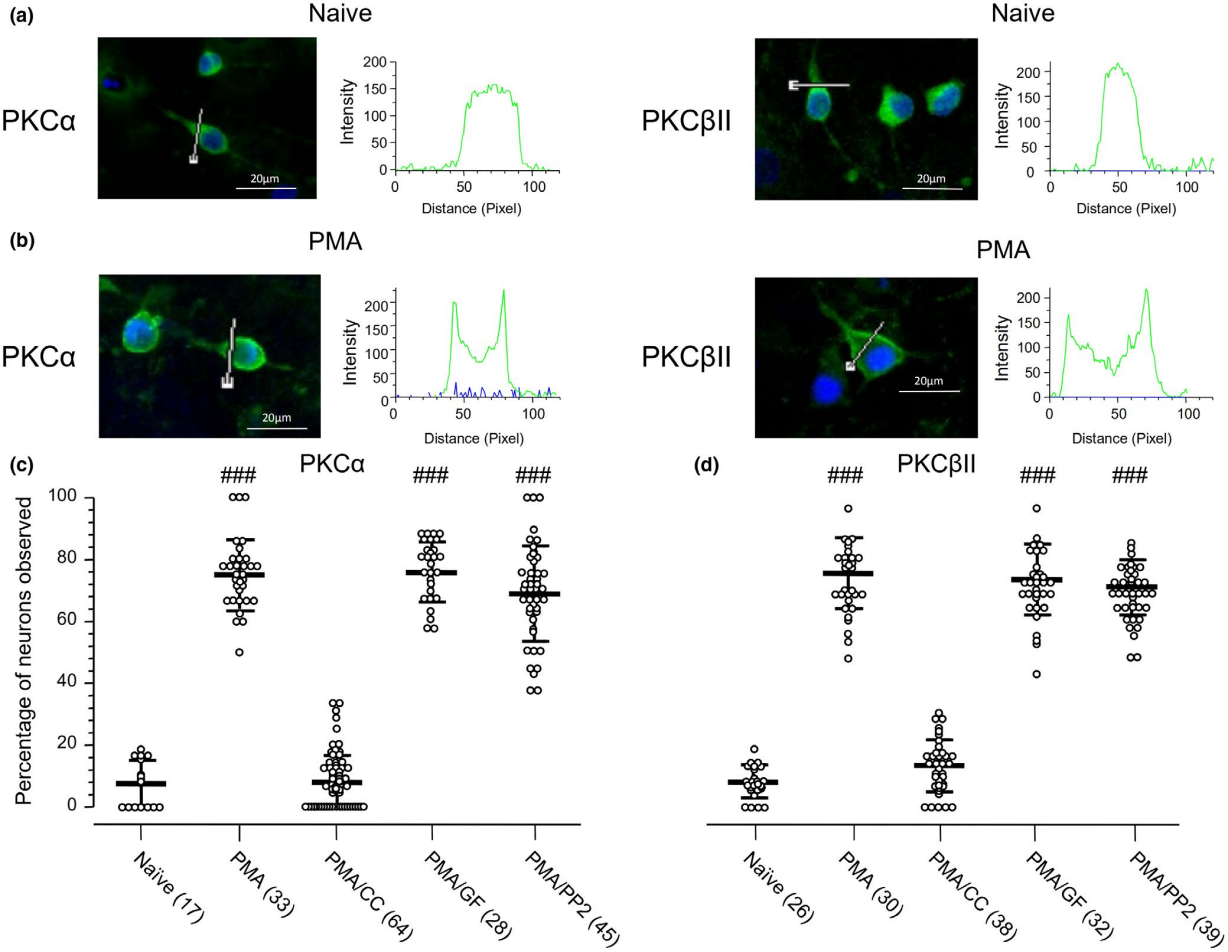
Distribution of PKCα and PKCβII in cultured arcuate nucleus (ARC) neurons. (a) Examples of DAPI (blue) co‐labeling with an antibody (green) against PKCα (RRID:AB_777294); upper) or PKCβII (RRID:AB_779042; lower) in naïve neurons; (b) The co‐labeling in PMA‐treated neurons. Fluorescent intensity plots [Blue: DAPI staining; Green: PKCα (RRID:AB_777294) or PKCβII (RRID:AB_779042) antibody staining] along the straight drawn lines are shown on the right of the images. (c) Percentages of observed neurons per field (mean ± *SD*), which displayed higher peak intensity staining of PKCα (RRID:AB_777294) antibody in both the 0%–20% and 80%–100% regions than that in the 20%–80% region of the line across neurons examined randomly. (d) Percentages of observed neurons per field (mean ± *SD*), which displayed higher peak intensity staining of PKCβII (RRID:AB_779042) antibody in both the 0%–20% and 80%–100% regions than that in the 20%–80% region of the line across neurons examined randomly. Naïve: neurons treated only with the culture medium; PMA: PMA application (10 μM for 30 min); PMA/CC: PMA application to neurons pre‐treated with chelerythrine chloride (CC) (10 μM) for 30 min; PMA/GF: PMA application to neurons pre‐treated with GF (5 μM) for 30 min; PMA/PP2: PMA application to neurons pre‐treated with PP2 (10 μM) for 30 min. ###: *p* < 0.001 Bonferroni's post hoc test in comparisons between the naïve and PMA‐treated neurons without or with a pre‐treatment of CC, GF, or PP2 for 30 min, in one‐way ANOVA (*p* < 0.0001, *F*_4,182_ = 380.4 for the panel (c); *p* < 0.0001, *F*_4,160_ = 425.1 for the panel (d)). Number of fields observed is indicated by values in brackets [Color figure can be viewed at https://www.wileyonlinelibrary.com]

Previous studies have shown that the activity of PKCs may be regulated by their subcellular localization (Farrar & Anderson, 1985; Farrar, Thomas, & Anderson, 1985; Mochly‐Rosen, 1995; Nishizuka, 1992), and that stimulating PKCs by application of PKC activators such as PMA may induce PKC translocation from the cytoplasm to the plasma membrane (Chao et al., 1998; Mochly‐Rosen, 1995; Nishizuka, 1992; Siomboing et al., 2001). It has also been found that PKC translocation may play an important role in the regulation of nociception (Gu et al., 2016; He & Wang, 2015). Since the expression of pPKCα/βII was found to be increased following PMA treatment (see Figure 6), the distribution of PKCα and PKCβII was examined in this work. Fluorescence images showing the distribution of PKCα and PKCβII were examined in 5.2 ± 0.2 (mean ± *SD*) fields per coverslip (*n* = 68 coverslips). Each field contained 11.3 ± 0.3 neurons (*n* = 355 fields). The peak intensities of fluorescent staining with PKCα (RRID:AB_777294) and PKCβII (RRID:AB_779042) antibodies were measured respectively for the 0%–20%, 20%–80%, and 80%–100% regions along the straight line across a neuron. If the peak intensities in both the 0%–20% and 80%–100% regions were higher than that in the 20%–80% region, an “enriched expression on the plasma membrane region” was then defined in this work. Since in some cases the line for measurement appeared in the perinucleus region, as indicated by DAPI staining (see Figure 7b), we conducted a correlation analysis between the intensities of PKCα or PKCβII antibody staining and DAPI staining for each of these cases. Since no statistically significant negative co‐relation was found in these cases [For an example: *Pearson r* = 0.0356, *p* = 0.8316, *R* ^2^ *=* 0.0013 for the intensities of PKCα antibody vs. DAPI staining recorded from the distances (Pixels) 42–79 along the straight line in the case shown in Figure 7b], the higher intensity staining detected on the plasma region was less likely due to the line drawing through the perinucleus region.

We found that in randomly examined untreated naïve neurons, 7.6% ± 1.8% (*n* = 17 fields; 208 neurons) and 8.6% ± 1.0% (*n* = 26 fields; 354 neurons) showed the enriched expressions of PKCα (RRID:AB_777294) and PKCβII (RRID:AB_779042) on the plasma membrane region, respectively (see Figure 7a, c, d). Following PMA treatment (10 μM for 30 min) the enriched expressions of PKCα (RRID:AB_777294) and PKCβII (RRID:AB_779042) on the plasma membrane region were found in 75.0% ± 2.0% (*n* = 33 fields; 313 neurons) and 75.9% ± 2.1% (*n* = 30 fields; 264 neurons) of randomly examined neurons (see Figure 7b, c, d). There were significant changes in the distribution of PKCα and PKCβII when compared with those in untreated naïve neurons (*p* < 0.001, Bonferroni's post hoc test following one‐way ANOVA [Figure 7c: *p* < 0.0001, *F*_4,182_ = 380.4; Figure 7d: *p* < 0.0001, *F*_4,160_ = 425.1]). However, the PMA application did not produce similar effects in neurons pre‐treated with CC (10 μM) for 30 min (see Figure 7c and d, Figure S3). The PMA‐induced changes in distributions of PKCα and PKCβII were not affected in neurons pre‐treated with GF (5 μM) or PP2 (10 μM) for 30 min (see Figures 7c,d and S3). This finding indicates that the regulations of the SFK and AP firing activities by PKCs in cultured ARC neurons are not dependent upon the translocation of PKCα and PKCβII.

## 4 DISCUSSION

**Figure 8:**
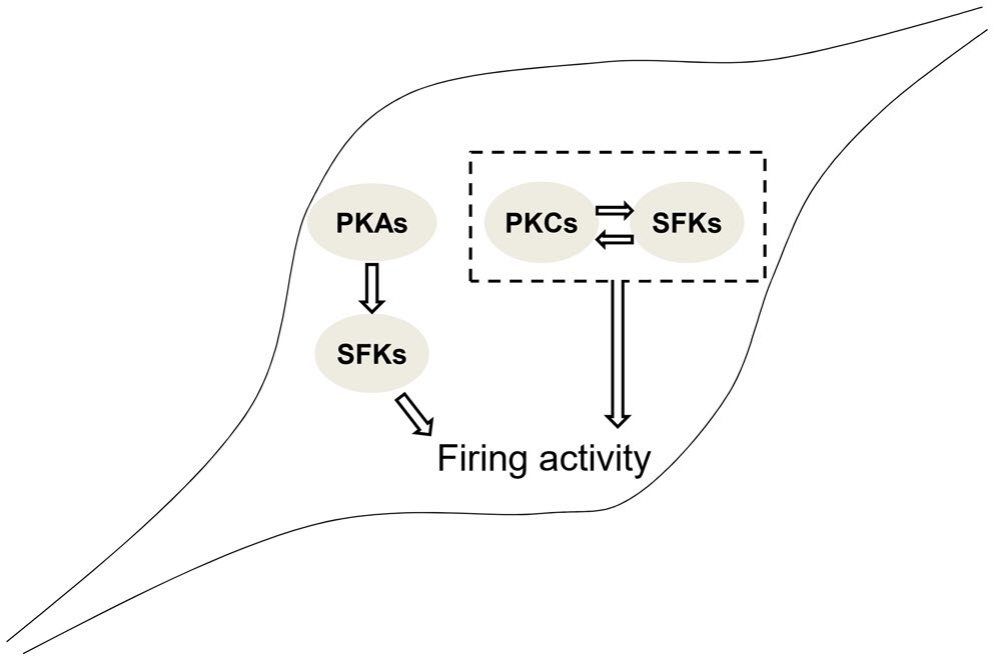
The regulation of the action potential (AP) firing activity by protein kinase A family (PKAs), protein kinase C family (PKCs), or Src family kinase (SFKs) in cultured arcuate nucleus (ARC) neurons. A diagram shows functional relationships between PKAs and SFKs or PKCs and SFKs in the regulation of the AP firing activity induced by depolarizing current injections [Color figure can be viewed at https://www.wileyonlinelibrary.com]

This study has identified that PKAs may act as an upstream factor(s) to enhance SFKs (see Figure 8) and that PKCs and SFKs may interact reciprocally—PKCs and SFKs may activate each other and the inhibition of either may abolish the up‐regulation of the AP firing activity induced by PKC‐SFK signaling in cultured ARC neurons (see Figure 8).

Although findings reported previously were not in consistence, many data have strongly suggested that protein tyrosine phosphatase alpha (PTPα) and C‐terminal Src kinases (Csks) are involved in the regulation of SFKs by PKCs (Benes & Soltoff, 2001; Kaul et al., 2005) or PKAs (Abrahamsen et al., 2003; Trepanier et al., 2013; Vang et al., 2001; Yang, Roselli, Patchev, Yu, & Almeida, 2013; Yaqub et al., 2003). PTPα specifically dephosphorylates the phosphorylated tyrosine residue in the C‐tails of SFKs (equivalent to pY527 of chicken c‐Src), and thereby activate SFKs (Brandt et al., 2003; Groveman et al., 2012; Lei et al., 2002; Thomas & Brugge, 1997; Zheng, Resnick, & Shalloway, 2000, 2002). Csks specifically phosphorylates the tyrosine residue (equivalent to Y527 in chicken c‐Src) in the C‐tails of SFKs, and thereby inhibit SFKs (Groveman et al., 2012; Nada et al., 1993; Okada, Nada, Yamanashi, Yamamoto, & Nakagawa, 1991; Xu et al., 2008). In addition, it has been also found that activated PKAs or PKCs may trigger the phosphorylation of SFKs such as Src (Yang et al., 2013). PKAs may also directly phosphorylate SFKs such as Fyn at S21, and thereby regulate focal adhesion targeting (Yeo et al., 2011) or cause the dissociation of Fyn and striatal‐enriched protein tyrosine phosphatase 61, and thereby activates Fyn (Yang et al., 2011). SFKs may phosphorylate PKCδ at Y311, and thereby increasing the activity of this kinase (Benes & Soltoff, 2001; Kaul et al., 2005). Conversely, inhibiting SFKs may lead to a depression of PKCδ activity (Kaul et al., 2005).

Controversially, however, it has also been reported that PKA may activate Csk by phosphorylating S364 of the kinase *in vitro* and *in vivo* (Vang et al., 2001; Yaqub et al., 2003), and thereby inhibit SFKs (Abrahamsen et al., 2003; Trepanier et al., 2013; Vang et al., 2001; Yaqub et al., 2003). Thus, PKA appears to act as an upstream factor which may activate (Sun et al., 1997; Yang et al., 2011; Yeo et al., 2011) or inhibit SFKs (Abrahamsen et al., 2003; Trepanier et al., 2013; Vang et al., 2001; Yaqub et al., 2003). Detailed molecular mechanisms underlying the reciprocal interaction between PKCs and SFKs and the regulation of SFKs by PKAs identified in cultured ARC neurons remain unclear. Thus, as the next step, characterizing detailed mechanisms underlying the functional interaction among PKAs, PKCs, and SFKs is still critically needed.

The AP firing activity of neurons is regulated by cation currents mediated by voltage‐gated Na^+^ and K^+^ channels. It is known that depending upon the subtypes of channels expressed, some Na^+^ (Ahern, Zhang, Wookalis, & Horn, 2005; Ahn, Beacham, Westenbroek, Scheuer, & Catterall, 2007; Beacham, Ahn, Catterall, & Scheuer, 2007) or K^+^ (Holmes, Fadool, Ren, & Levitan, 1996; Li, Langlais, Gamper, Liu, & Shapiro, 2004; Sobko, Peretz, & Attali, 1998; Strauss et al., 2002) channels may be up‐ or down‐regulated by SFKs. We have previously reported that voltage‐gated Na^+^ currents are regulated by endogenous SFKs in cultured cochlear spiral ganglion neurons (Feng et al., 2012). The inhibition of SFKs shifts the steady‐state inactivation curves of Na^+^ currents and delays the recovery of Na^+^ currents from inactivation, while activation of SFKs causes the left shift of the activation curve (Feng et al., 2012). Although detailed mechanisms still need to be clarified, the present study has documented that PKAs and PKCs may regulate the AP firing activity via SFKs (Figure 8).

Our *in vivo* studies have shown that knockdown of Src in the ARC area or systemic application of a SFK inhibitor does not induce any significant change in the sensitivity of normal rats while inflammation‐induced pain hypersensitivity is significantly diminished by Src knockdown in the ARC area or the systemic application of the SFK inhibitor (Ma et al., 2019). In this work, we found that although the increases in both the AP firing activity and the expression of phosphorylated PKAs, PKCs, or SFKs following the application of FSK or PMA could be prevented by application of the inhibitors of these kinases, under normal basal conditions no significant effect were found following the applications the PKA, PKC, and SFK inhibitors. Thus, further clarifying how these enzymes are activated in cultured ARC neurons as well as *in vivo* may reveal novel insights for understanding the signaling mediated by these kinases in the CNS.

Potential functional interactions among PKAs, PKCs, and SFKs have been implicated in the regulation of synaptic functions. For example, the activation of PKCs may up‐regulate the tyrosine phosphorylation of NMDARs (Grosshans & Browning, 2001) and the NMDAR‐mediated synaptic currents (Lu et al., 1999) through PTPα. We have previously found that the inhibition of PKCs or SFKs in the ARC area not only blocks the enhancement of expressions of active PKCs, SFKs, and phosphorylated NMDA GluN2B at Y1472 in this area, but also attenuates the inflammation‐induced increases in the discharge activity of ARC neurons and pain hypersensitivity (Bu et al., 2015; Peng et al., 2011; Xu et al., 2012; Zheng et al., 2016). Taken together with our present findings, it has been implicated that the functional interactions among these enzymes may be novel mechanisms involved in the regulation of neuronal excitability.

We have identified that in the ARC area Src, but not Fyn or Lyn in SFKs, is activated following the development of peripheral inflammation, and that Src knockdown in this area blocks the inflammation‐induced increases in the expressions of activated SFKs and the phosphorylated GluN2B subunit at Y1472 in the ARC area, and reduces pain hypersensitivity (Ma et al., 2019). Thus, studies focusing on whether and how the functional interactions among PKAs, PKCs, and SFKs identified in cultured ARC neurons are involved in the regulation of pain hypersensitivity *in vivo* are essential for us to understand mechanisms underlying the formation and maintenance of pain hypersensitivity. The findings that PKCs and SFKs may activate each other and the inhibition of either may abolish the up‐regulation of the AP firing activity by the PKC‐SFK signaling in cultured ARC neurons suggest a new avenue for developing novel approaches to treat increased excitability associated with pain hypersensitivity.

## CONFLICT OF INTEREST

The authors have no conflict of interest to declare.

## AUTHOR CONTRIBUTIONS

All authors have full access to all the data in the study and take responsibility for the integrity of the data and the accuracy of data analysis. *Conceptualization*: X.J., X.‐M.Y., and X.‐D.S.; *Methodology*: P.M.; *Formal Analysis*, X.‐D.S., A.W., P.M., and X.‐M.Y; *Investigation*, X.‐D.S., A.W., and P.M.; *Resources*, S.G.; *Data curation*, S.G., X.J., and P.M.; *Writing—original draft*, X.‐M.Y. and X.‐D.S.; *Writing—review editing*, X.‐M.Y., X.‐D.S., X.J., and J.T.; *Supervision*, X.J., X.‐M.Y., and J.T.; *Project administration*, X.J., X.‐M.Y., J.T., and S.G.; *Funding acquisition*, X.J. and J.T.

## Supporting information


**Figure S1.** The ARC region dissected for dissociated cell culture. The image shows ventral part of a coronal brain section (4 μm) cut with microtome (Leica, RM2235, Nussloch, Germany) from an 1‐day‐old Sprague‐Dawley rat pup and stained with H&E. Tissues in the ARC area as indicated within the dashed triangle were dissected for dissociated cell culture

**Figure S2.** Examples of original blots from the same full length PVDF membranes. (a) The gel (from left to right) was loaded with the prestained protein ladder (Thermo Scientific) and lysates respectively prepared from cultured ARC cells without any treatment (Naïve) and with treatment of FSK (50 μM). The PVDF membrane was stripped and successively probed (from top to bottom) with antibodies against pPKAs (RRID:AB_1524202), PKAs (RRID:AB_2750616), pSFKs (RRID:AB_10860257) and β‐actin (RRID:AB_2687938) as indicated. (b) The gel (from left to right) was loaded with the prestained protein ladder (Thermo Scientific) and lysates respectively prepared from cultured ARC cells without any treatment (Naïve) or treated with FSK (50 μM). The PVDF membrane was stripped and successively probed (from top to bottom) with pPKCα/βII (RRID:AB_2284224), pPKCpan (RRID:AB_2168219) and β‐actin (RRID:AB_2687938) antibodies as indicated. Parts of the blots are shown in Figure 5A

**Figure S3.** PMA‐induced redistribution of PKCα and PKCβII in cultured ARC neurons. (a) Examples of DAPI (blue) co‐labeling with an antibody against PKCα (RRID:AB_777294, top image) or PKCβII (RRID:AB_779042, bottom image) following PMA (10 μM) application to neurons pre‐treated with CC (10 μM) for 30 min; (b) Examples of DAPI (blue) co‐labeling with an antibody against PKCα (RRID:AB_777294, top) or PKCβII (RRID:AB_779042, bottom) following PMA (10 μM) application to neurons pre‐treated with GF (5 μM) for 30 min; (c) Examples of DAPI (blue) co‐labeling with an antibody against PKCα (RRID:AB_777294, top) or PKCβII (RRID:AB_779042, bottom) following PMA (10 μM) application to neurons pre‐treated with PP2 (10 μM) for 30 min

**Figure S4.** Effects of FSK or PMA application on the expression of the proteins PKAs, pPKAs, Src, PKCα, and PKCβII in cultured ARC cells. (a) Relative changes in the amount of proteins Src, PKAs, and pPKAs. The gels were loaded with lysates prepared from cultured ARC cells without any treatment (naïve) or treated with FSK (100 μM) and IBMX (50 μM) for 30 min. Each group of blots was cropped from the same PVDF membrane, stripped and successively probed with antibodies as indicated on the left of blots. The scatter graph shows summary data (mean ± *SD*) of relative changes in the expression of Src and PKAs, and in the ratio of pPKAs versus total PKAs. $: *p* < 0.05, unpaired *t* test in comparison with that in naïve cells (= 1, dashed line). (b) Relative changes in the amount of proteins PKCα, PKCβII, and Src. The gel was loaded with lysates prepared from cultured ARC cells without any treatment (naïve) or treated with PMA (10 μM) for 30 min. Each group of blots was cropped from the same PVDF membrane, stripped, and successively probed with antibodies as indicated on the left of blots. The scatter graph shows summary data (mean ± *SD*) of relative changes in the expression of PKCα, PKCβII, and Src

## Data Availability

All materials, data, and associated protocols are available to readers without undue qualifications in material transfer agreements.
