# Supplementary figures and images for: Regulation of the firing activity by PKA‐PKC‐Src family kinases in cultured neurons of hypothalamic arcuate nucleus
Source: J Neurosci Res. 2019 Aug 12;98(2):384–403. doi: 10.1002/jnr.24516 (PMC6916362; doi:10.1002/jnr.24516)

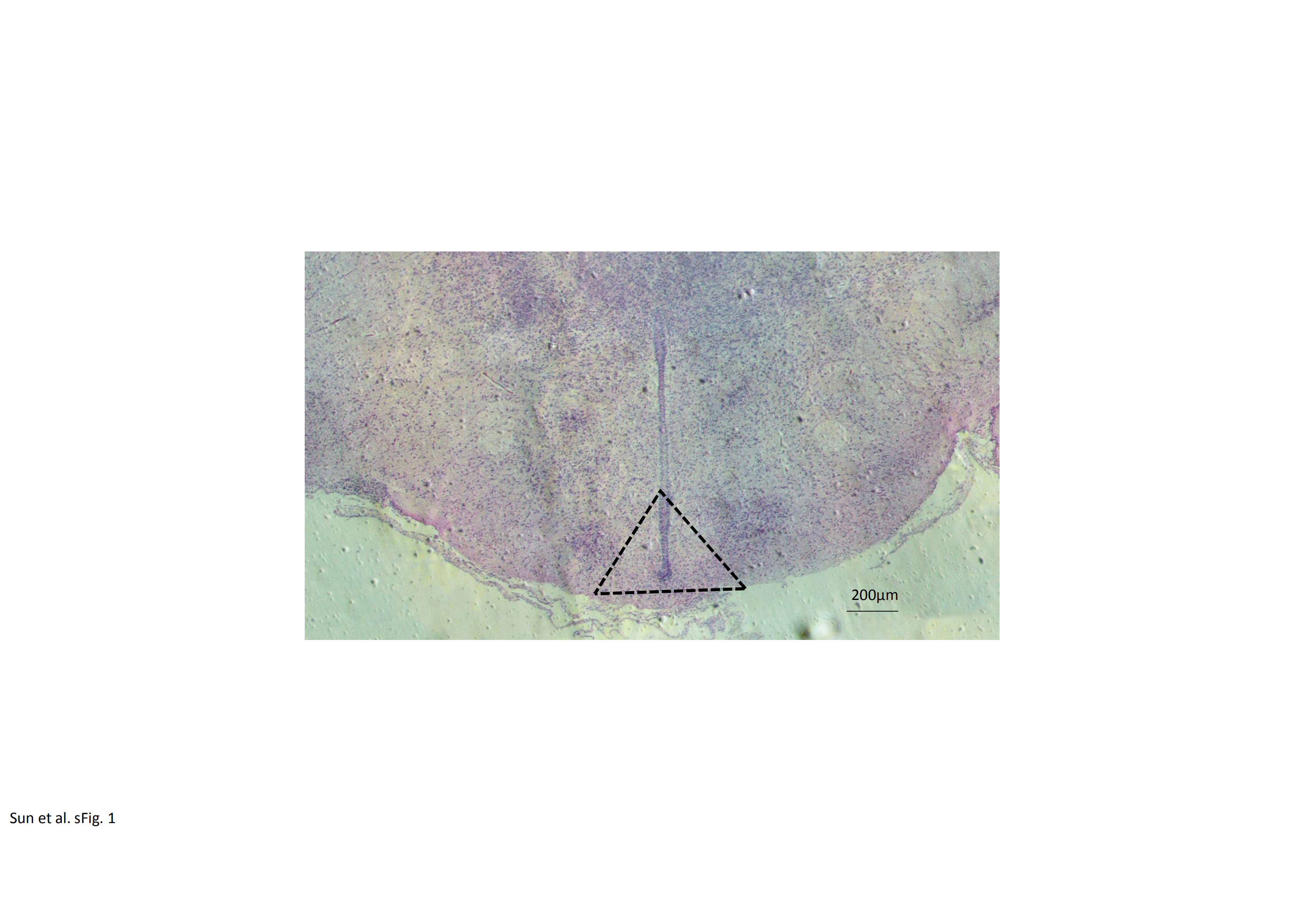

Supplement: Supplementary file 1 — Figure S1. The ARC region dissected for dissociated cell culture. The image shows ventral part of a coronal brain section (4 μm) cut with microtome (Leica, RM2235, Nussloch, Germany) from an 1‐day‐old Sprague‐Dawley rat pup and stained with H&E. Tissues in the ARC area as indicated within the dashed triangle were dissected for dissociated cell culture [file JNR-98-384-s001.tif]

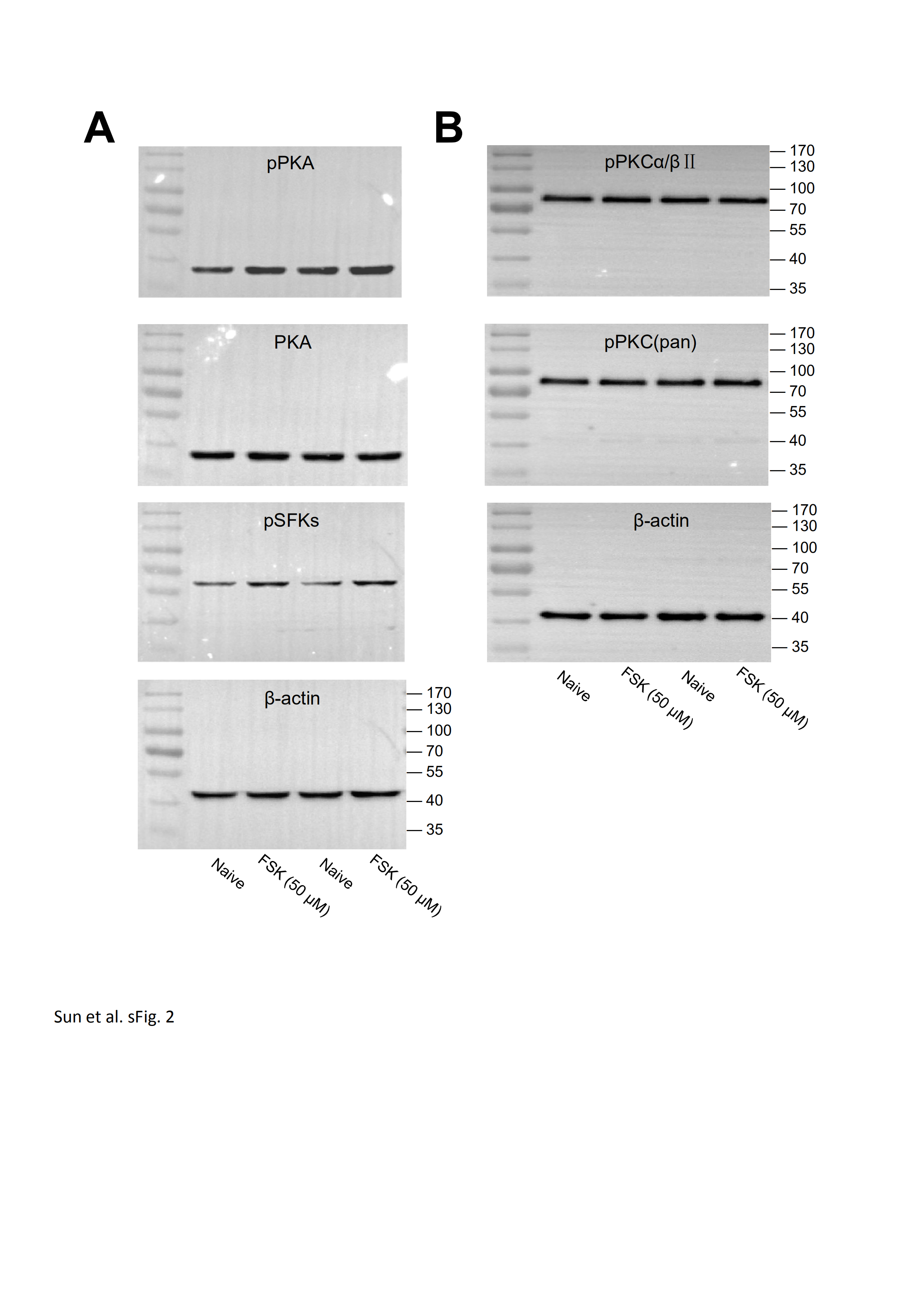

Supplement: Supplementary file 2 — Figure S2. Examples of original blots from the same full length PVDF membranes. (a) The gel (from left to right) was loaded with the prestained protein ladder (Thermo Scientific) and lysates respectively prepared from cultured ARC cells without any treatment (Naïve) and with treatment of FSK (50 μM). The PVDF membrane was stripped and successively probed (from top to bottom) with antibodies against pPKAs (RRID:AB_1524202), PKAs (RRID:AB_2750616), pSFKs (RRID:AB_10860257) and β‐actin (RRID:AB_2687938) as indicated. (b) The gel (from left to right) was loaded with the prestained protein ladder (Thermo Scientific) and lysates respectively prepared from cultured ARC cells without any treatment (Naïve) or treated with FSK (50 μM). The PVDF membrane was stripped and successively probed (from top to bottom) with pPKCα/βII (RRID:AB_2284224), pPKCpan (RRID:AB_2168219) and β‐actin (RRID:AB_2687938) antibodies as indicated. Parts of the blots are shown in Figure 5A [file JNR-98-384-s002.tif]

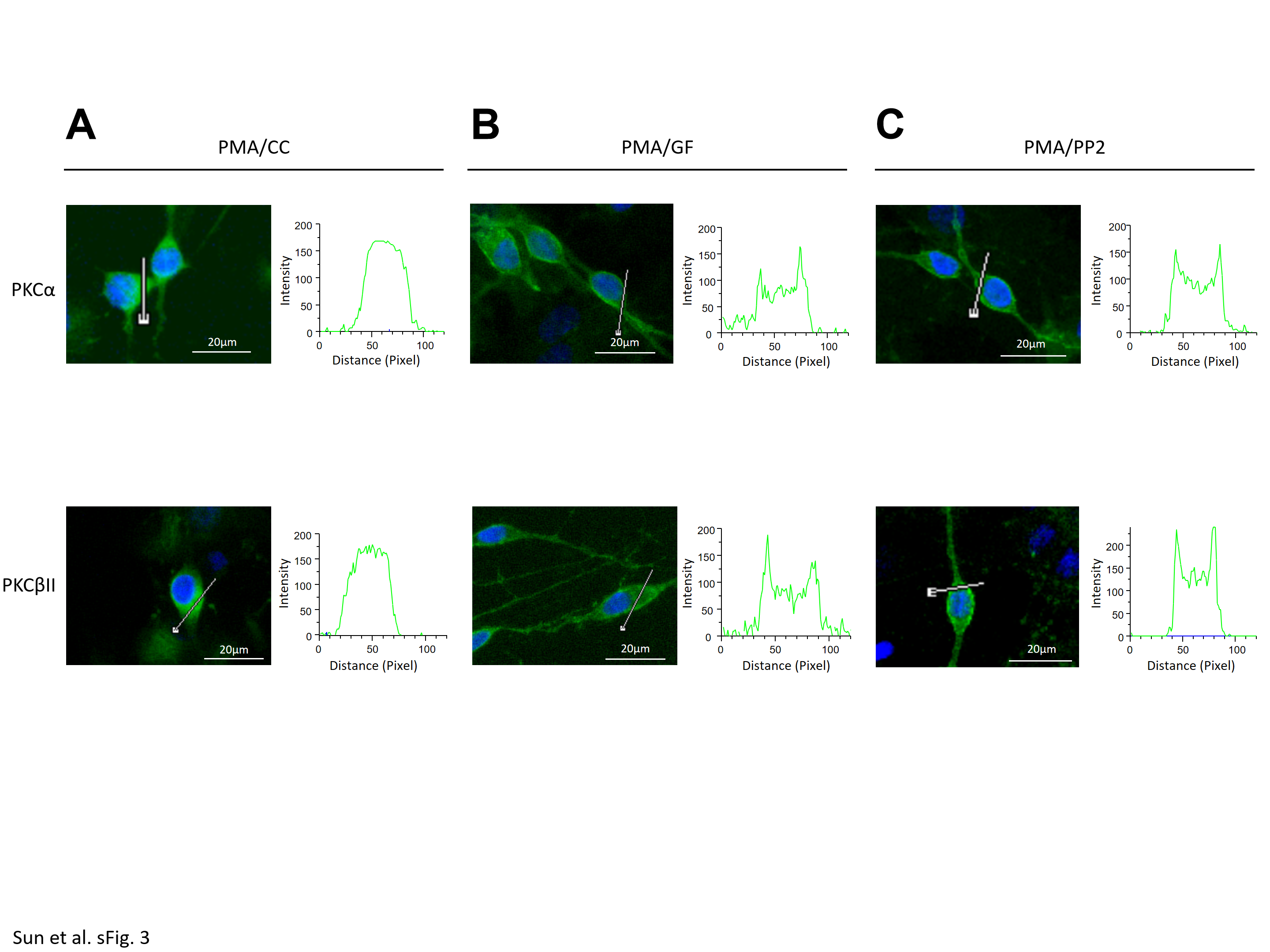

Supplement: Supplementary file 3 — Figure S3. PMA‐induced redistribution of PKCα and PKCβII in cultured ARC neurons. (a) Examples of DAPI (blue) co‐labeling with an antibody against PKCα (RRID:AB_777294, top image) or PKCβII (RRID:AB_779042, bottom image) following PMA (10 μM) application to neurons pre‐treated with CC (10 μM) for 30 min; (b) Examples of DAPI (blue) co‐labeling with an antibody against PKCα (RRID:AB_777294, top) or PKCβII (RRID:AB_779042, bottom) following PMA (10 μM) application to neurons pre‐treated with GF (5 μM) for 30 min; (c) Examples of DAPI (blue) co‐labeling with an antibody against PKCα (RRID:AB_777294, top) or PKCβII (RRID:AB_779042, bottom) following PMA (10 μM) application to neurons pre‐treated with PP2 (10 μM) for 30 min [file JNR-98-384-s003.tif]

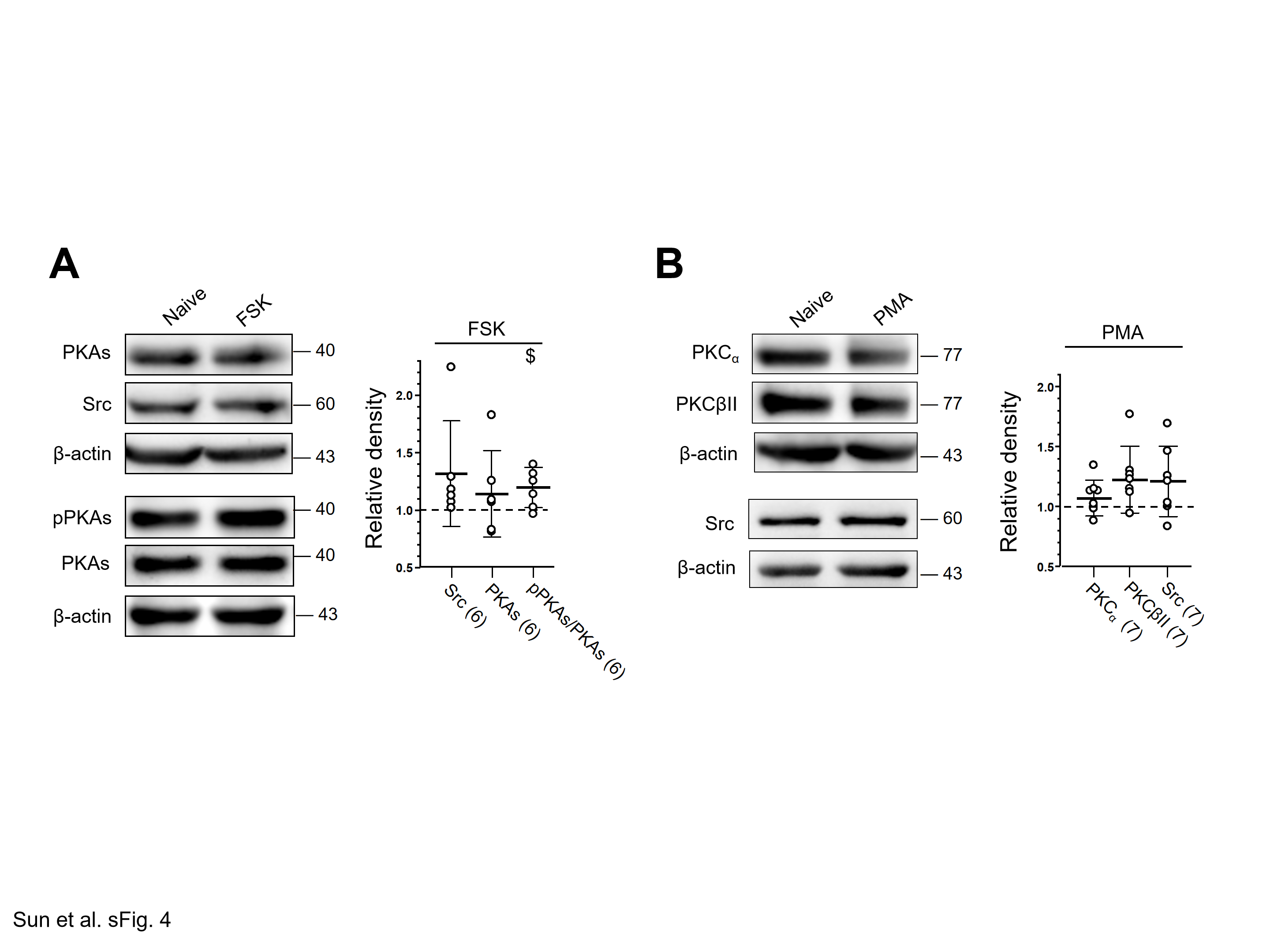

Supplement: Supplementary file 4 — Figure S4. Effects of FSK or PMA application on the expression of the proteins PKAs, pPKAs, Src, PKCα, and PKCβII in cultured ARC cells. (a) Relative changes in the amount of proteins Src, PKAs, and pPKAs. The gels were loaded with lysates prepared from cultured ARC cells without any treatment (naïve) or treated with FSK (100 μM) and IBMX (50 μM) for 30 min. Each group of blots was cropped from the same PVDF membrane, stripped and successively probed with antibodies as indicated on the left of blots. The scatter graph shows summary data (mean ± SD) of relative changes in the expression of Src and PKAs, and in the ratio of pPKAs versus total PKAs. $: p < 0.05, unpaired t test in comparison with that in naïve cells (= 1, dashed line). (b) Relative changes in the amount of proteins PKCα, PKCβII, and Src. The gel was loaded with lysates prepared from cultured ARC cells without any treatment (naïve) or treated with PMA (10 μM) for 30 min. Each group of blots was cropped from the same PVDF membrane, stripped, and successively probed with antibodies as indicated on the left of blots. The scatter graph shows summary data (mean ± SD) of relative changes in the expression of PKCα, PKCβII, and Src [file JNR-98-384-s004.tif]
